# Discovery of EMRE in fungi resolves the true evolutionary history of the mitochondrial calcium uniporter

**DOI:** 10.1038/s41467-020-17705-4

**Published:** 2020-08-12

**Authors:** Alexandros A. Pittis, Valerie Goh, Alberto Cebrian-Serrano, Jennifer Wettmarshausen, Fabiana Perocchi, Toni Gabaldón

**Affiliations:** 1 Centre for Genomic Regulation (CRG), The Barcelona Institute of Science and Technology, 08003 Barcelona, Spain; 2 Universitat Pompeu Fabra (UPF), 08003 Barcelona, Spain; 3 Biodiversity Research Centre & Department of Botany, University of British Columbia (UBC), Vancouver, BC V6T 1Z4 Canada; 4 Institute for Diabetes and Obesity, Helmholtz Diabetes Center (HDC), Helmholtz Zentrum München, 85764 Neuherberg, Germany; 5 German Center for Diabetes Research (DZD), 85764 Neuherberg, Germany; 6 Munich Cluster for Systems Neurology, 81377 Munich, Germany; 7 ICREA, Pg. Lluís Companys 23, 08010 Barcelona, Spain; 8 Present Address: Berlin Institute for Advanced Study, 14193 Berlin, Germany; 9 Present Address: Barcelona Supercomputing Centre (BSC-CNC) and Institute for Research in Biomedicine (IRB), 08034 Barcelona, Spain

**Keywords:** Mitochondria, Phylogenetics, Eukaryote, Phylogenomics

## Abstract

Calcium (Ca^2+^) influx into mitochondria occurs through a Ca^2+^-selective uniporter channel, which regulates essential cellular processes in eukaryotic organisms. Previous evolutionary analyses of its pore-forming subunits MCU and EMRE, and gatekeeper MICU1, pinpointed an evolutionary paradox: the presence of MCU homologs in fungal species devoid of any other uniporter components and of mt-Ca^2+^ uptake. Here, we trace the mt-Ca^2+^ uniporter evolution across 1,156 fully-sequenced eukaryotes and show that animal and fungal MCUs represent two distinct paralogous subfamilies originating from an ancestral duplication. Accordingly, we find EMRE orthologs outside Holoza and uncover the existence of an animal-like uniporter within chytrid fungi, which enables mt-Ca^2+^ uptake when reconstituted in vivo in the yeast *Saccharomyces cerevisiae*. Our study represents the most comprehensive phylogenomic analysis of the mt-Ca^2+^ uptake system and demonstrates that MCU, EMRE, and MICU formed the core of the ancestral opisthokont uniporter, with major implications for comparative structural and functional studies.

## Abstract (web summary)

The mitochondrial calcium uptake system, crucial for cellular processes, evolved in ancient eukaryotes. Here, authors perform a phylogenomic analysis across 1,156 eukaryotes, and show that previously identified animal and fungal genes in this system originated from an ancestral duplication.

## Introduction

Mitochondria from several organisms play a key role in regulating intracellular Ca^2+^ signaling^1^ due to their ability to rapidly uptake and buffer cytosolic Ca^2+^. Mitochondrial Ca^2+^ (mt-Ca^2+^) uptake is mediated by a highly selective channel, the mt-Ca^2+^ uniporter, that resides in the inner mitochondrial membrane and is powered by the negative transmembrane potential^2–4^. However, the functional role of mt-Ca^2+^ uptake and its pathophysiology has remained largely unknown, as the molecular identity of the mt-Ca^2+^ uniporter channel was only recently discovered^5–7^. To this end, comparative genomics analyses based on a few eukaryotic species, in combination with RNAi assays, were instrumental in identifying the first components of the mammalian uniporter, MCU and MICU1 (refs. ^5–7^). While MCU constitutes the pore-forming and Ca^2+^-conducting subunit of the uniporter^8^, the EF-hand-containing and Ca^2+^-sensitive protein MICU1 has been shown to function as a channel gatekeeper and cooperative activator^9^. Importantly, their discovery paved the way to the identification of additional structural and regulatory components of the mammalian uniporter, including a MCU-dominant-negative beta subunit (MCUb)^10^, three tissue-specific MICU1 paralogs or splice variants (MICU2 (refs. ^11,12^), MICU3 (ref. ^13^), and MICU1.1 (ref. ^14^)), and an essential MCU regulator (EMRE)^15^. When co-expressed with MCU, EMRE was shown to be necessary and sufficient to form a functional Ca^2+^ channel and to reconstitute mammalian-like uniporter activity even in yeast mitochondria^15,16^, which are otherwise incapable of Ca^2+^ uptake^16–18^.

Altogether, these findings have highlighted a complex multimeric nature for the mammalian uniporter, whose composition, stoichiometry, and regulation need to be fine-tuned to the physiological demands of each cell and tissue. However, the functional and mechanistic role of each uniporter component and the molecular basis of their interdependence are still unclear. Furthermore, although several observations have pointed to an ancient eukaryotic origin of mt-Ca^2+^ uptake, the identification of several organisms with a different uniporter’s composition in different clades have raised interest in understanding the structural basis of uniporter activity^19–24^. For example, while MCU and MICU1 showed highly correlated evolutionary histories across 138 sequenced eukaryotic organisms, EMRE apparently lacked any homolog outside the metazoan lineage and was therefore suggested to be an animal-specific innovation^25,26^. Consistently, *Dictyostelium discoideum*, an amoebozoan that diverged earlier than the origin of opisthokonts, expresses both MCU and MICU1 orthologs, which form a functional uniporter in the absence of EMRE^16^. Despite the ancient origin of their interaction and the overall observed high co-evolution between MCU and MICU1, most fungi represented an exception to this rule, with most fungal species encoding homologs of MCU but not of MICU1 (refs. ^25,26^). The identification of MCU as the only uniporter component in Basidiomycota and filamentous Ascomycota (e.g., *Neurospora crassa* and *Aspergillus fumigatus*) has been described as an evolutionary paradox (see ref. ^27^), but has also been interpreted as indicating that fungal MCUs could be sufficient for mt-Ca^2+^ uptake and regulated independently of MICU1 (refs. ^25,26^). Based on the assumption of an orthologous relationship between human and fungal MCUs^25,26,28^, several independent structural studies of Ascomycota MCUs have been performed to understand the basic principles of uniporter channel assembly and function^21–24^. However, those organisms had been shown to lack uniporter activity^29,30^ and their MCU homologs were unable to mediate mt-Ca^2+^ uptake when heterologously expressed in HeLa or yeast cells^17,24^. Not surprisingly, significant structural and sequence differences were found between fungal MCUs and their animal counterparts^20–24^, raising the question of whether fungal MCUs function as classical Ca^2+^ uniporters at all.

Here, we perform a comprehensive evolutionary analysis of the mt-Ca^2+^ uniporter and show that a gene duplication at the opisthokont common ancestor of animals and fungi resulted in two distinct MCU paralogous clades, differentially retained in the two groups. When only the animal-like clade is considered, we observe fully consistent co-evolutionary patterns between MCU and MICU (MICU1’s family), across eukaryotes, and of these with EMRE, across opisthokonts. We find that only three early diverging fungi contain both the fungal paralog and the complete animal-like MCU complex (MCU, MICU, and EMRE). Consistently, we find that the heterologous expression of MCU and EMRE from fungal species with an animal-like MCU complex results in the functional reconstitution of Ca^2+^ uptake in mitochondria of HeLa and yeast cells. Conversely, representatives of the fungal paralogs do not show uniporter activity, suggestive of an alternative function or of the requirement for a yet unidentified regulator. Altogether, our phylogenomic and functional analyses of the mt-Ca^2+^ uptake system demonstrate that MCU, EMRE, and MICU represent the minimal core components of the ancestral opisthokont uniporter and pinpoint key targets for comparative structural and functional studies. Finally, this study also confirms the importance and power of thorough evolutionary analyses to understand the molecular basis of functional interactions within a protein complex.

## Results

### The mitochondrial calcium uniporter evolution

**Fig. 1:**
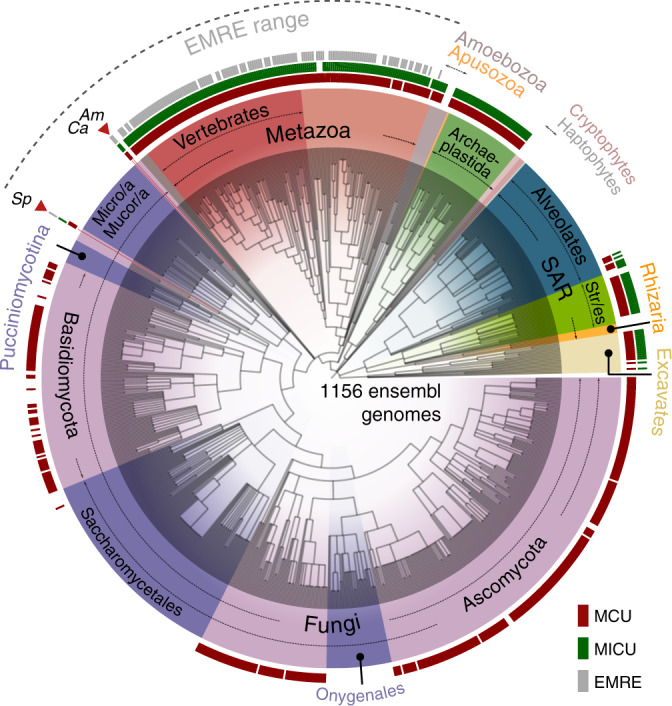
Phylogenetic distribution of mt-Ca^2+^ uniporter protein families. The phylogenetic distribution of MCU (red), MICU (green), and EMRE (gray) homologs across 1156 eukaryotic genomes is shown on the NCBI taxonomy tree. Viridiplantae and Rhodophyta (red algae) have been grouped together as Archaeplastida, and alveolates, stramenopiles (Str/es), and rhizarians as the SAR clade. In all cases where data from various strains of a species are present with the same pattern, these have all been collapsed to the species level, resulting in 969 terminal nodes shown. The mt-Ca^2+^ uniporter complex has been completely lost in Apicomplexa within Alveolates, Rhizaria (5 genomes), red algae (3 genomes), Cryptophytes (3 genomes), Haptophytes (1 genome), and the Entamoeba clade within Amoebozoa. Within fungi (in purple), all major clades that have completely lost MCU homologs are indicated with a darker purple color, namely Onygenales, Saccharomycetales, Pucciniomycotina, Mucoromycotina (Mucor/a), and Microsporidia (Micro/a). The only three early diverging fungal species (*A. macrogynus-Am*, *C. anguillae-Ca*, *S. punctatus-Sp*) that encode also MICU and EMRE are highlighted with a red arrowhead. The NCBI taxonomy and the presence/absence profile were visualized using the ETE toolkit^41^. For a version of the profile, which includes the species names, see Supplementary Fig. 1.

We assessed the evolution of each uniporter component across 1156 fully-sequenced eukaryotic genomes (see Supplementary Data 1), using a combination of profile-based sequence searches, protein domain composition assessment, and phylogenetics. As shown in Fig. 1 (Supplementary Fig. 1), the overall taxonomic distributions of MCU and MICU1 homologs were largely congruent with that of previous genomics surveys^25,26^. We confirmed the presence of MCU in at least some species of the major eukaryotic groups (Unikonts, the SAR clade, Plants, and Euglenozoa), and its absence in all sequenced Apicomplexans, Microsporidia, *Trichomonas*, and *Giardia*, and all yeasts in Saccharomycotina and most in *Schizosaccharomyces*,. Hence, mt-Ca^2+^ uptake appeared to have been lost many times independently during the evolution of eukaryotes. A significant number of these losses correlated with extreme streamlining of mitochondrial metabolism, as most MCU/MICU-lacking lineages encompassed relict forms of anaerobic mitochondria, such as mitosomes (microsporidians, *Entamoeba*, *Giardia, Cryptosporidium*) or hydrogenosomes (*Trichomonas*)^31^. Our homology-based results confirmed the above-mentioned anomaly that most fungal genomes, for which our dataset is particularly rich—776 species as compared to 50 in previous studies^25,26^—encode for MCU but not MICU or EMRE. Unexpectedly, our analysis uncovered the presence of EMRE outside Holozoa, identifying reliable orthologs in three chytrid fungi—an early diverging zoosporic fungal lineage: *Allomyces macrogynus*, *Catenaria anguillulae*, and *Spizellomyces punctatus*. Additional searches in public databases confirmed that EMRE was not present in other sequenced fungi.

**Fig. 2:**
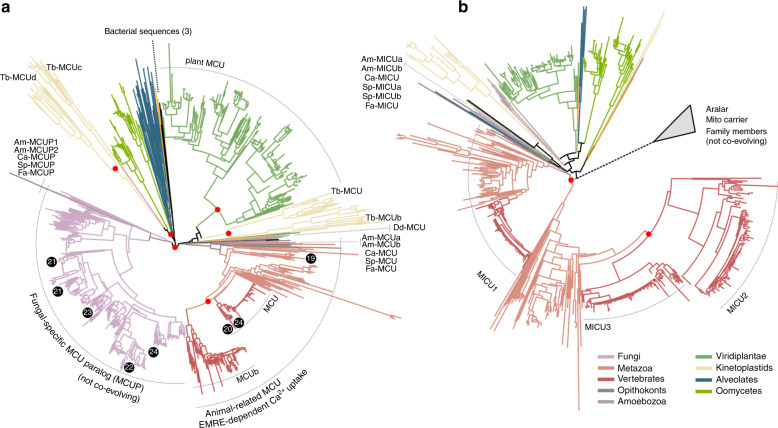
The fungal-specific MCU is a paralogous distinct phylogenetic clade. Maximum Likelihood phylogenetic trees of MCU (**a**) and MICU (**b**) families. The two families have been expanded through duplication rounds in various independent lineages. Major duplication events are indicated with a red sphere on the relevant tree node. The co-occurrence between the two families is almost perfect, if only the true orthologous sequences are considered. MCUP (fungal-specific MCU paralog) and Aralar (MICU distant homolog) sequences are not co-evolving with MCU and MICU, respectively (see also Methods). In both **a** and **b** names and relative positions in the trees of members from Holomycota and few other representative species are shown. The main sub-families (MCU/MCUb and MICU1/2/3) are named after the human/mouse sequences within the phylogenetic clades. The phylogenetic positions of MCUs or MCUPs from published structural data are shown in black circles, and the numbers refer to the publication reference. The number of bacterial sequences included in the tree is indicated in brackets. The raw phylogenetic trees in newick format are provided in Supplementary Data 2.

To clarify the underlying evolutionary history of the uniporter, we reconstructed and inspected the molecular phylogenies of MCU (Fig. 2a, Supplementary Fig. 2a) and MICU1 (Fig. 2b, Supplementary Fig. 2b) homologs across eukaryotes. We found that the evolution of both MCU and MICU gene families was driven by numerous gene duplications and losses, some of them having occurred in parallel in different lineages, implying an ancient and tight functional relationship. Furthermore, we showed that evolutionary-independent duplications at the base of several main eukaryotic lineages—vertebrates, streptophytes, oomycetes, kinetoplastids, and ciliates—resulted in the existence of multiple MCU paralogous copies in each of these clades. As a result, orthology relationships within the MCU gene family are complex and of the type many-to-many^32^. This means, for instance, that human MCU and MCUb are equally distant evolutionarily (co-orthologous) to each non-vertebrate animal MCUs, and also to all MCUs from protists and plants, explaining why *Trypanosoma*’s MCUb does not functionally complement human MCUb^33,34^. It also implies that shared physical interactions between paralogous MCU proteins in hetero-oligomers in different organisms, as shown for human and *Trypanosoma brucei* MCUb proteins^10,33,34^, are the result of parallel evolution. Importantly, one duplication event in the MCU gene family occurred in the common ancestor of opisthokonts and was followed by differential losses that distinguished Holomycota (fungi and their relatives, including *Fonticula alba*) from the other opisthokonts, i.e. the Holozoa (animals and their unicellular close relatives). Most fungal species kept only one of the two MCU paralogs that is referred here as the fungal-specific MCU paralog (MCUP). Holozoa, instead, retained the other MCU paralog, the bona-fide animal MCU. Only three chytrid fungi in our dataset, *A. macrogynus*, *C. anguillulae*, and *S. punctatus* retained both the MCUP and the animal MCU, and these are also the only fungi encoding MICU1 and EMRE homologs. This striking, previously undetected, co-evolution pattern between MCU, MICU, and EMRE in chytrids suggests a strong interdependence, and even stronger considering that Blastocladiales (*Allomyces* and *Catenaria*) and *Spizellomyces* do not form a monophyletic clade^35^. Based on these findings, we hypothesized that these animal-like MCUs present in chytrids should require EMRE to drive mt-Ca^2+^ uptake, similarly to their human ortholog.

### Reconstitution of mt-Ca^2+^ uptake by chytrid MCU and EMRE

**Fig. 3:**
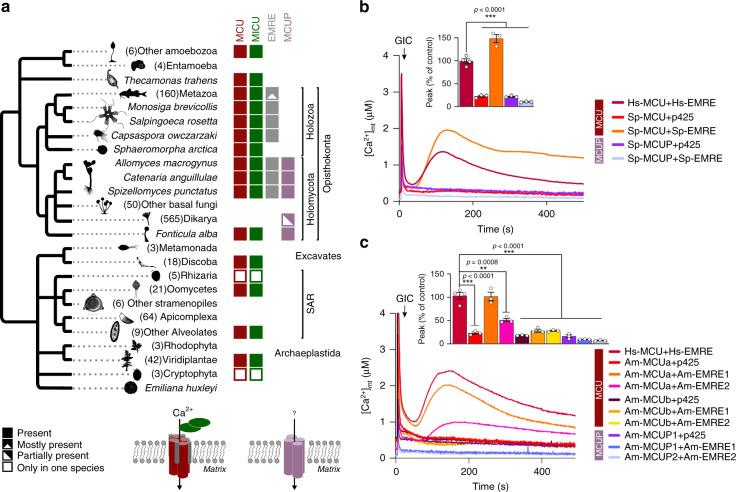
Functional reconstitution of mt-Ca^2+^ uptake by fungal MCU and EMRE homologs. **a** Phylogenetic distribution profile (presence/absence) across MCU complex components. The distribution pattern of MICU (across eukaryotes) and EMRE (in opisthokonts) largely overlaps with that of the animal-like MCU, but not the MCUP. **b**, **c** Representative traces and quantification of mt-Ca^2+^ transients in yeast cells expressing human MCU and EMRE (*n* = 4) or animal-like or fungal-specific MCU orthologs from *S. punctatus* (*n* = 3) (**b**) and *A. macrogynus* (*n* = 3) (**c**) with either their respective EMRE proteins or empty vector (p425) upon glucose-induced calcium (GIC) stimulation in presence of 1 mM CaCl_2_. All data represent mean ± SEM. *P* values are indicated in the different panels (**b**, **c** ***p < 0.0001, one-way ANOVA with Dunnett’s multiple comparisons test). The silhouette images in **a** representing the different lineages were downloaded from PhyloPic (http://phylopic.org/) or wikipedia (https://www.wikipedia.org/), or designed by A.A.P. in Inkscape (https://inkscape.org/). All downloaded images were available for reuse under a Public Domain license and do not require attribution. Source data of **b** and **c** are provided as a Source Data file.

The above-mentioned finding of bona-fide EMRE orthologs in these three chytrids (Fig. 1) placed back the origin of an animal-like mt-Ca^2+^ uptake in the opisthokont ancestor, preceding the diversification of animals and fungi. Consistently, the heterologous expression of MCU from *Dictyostelium discoideum*, representing an amoebozoan lineage that diverged earlier than the origin of opisthokonts, is alone sufficient to reconstitute mt-Ca^2+^ uptake in yeast mitochondria, while human (Hs-) MCU only does so in the presence of EMRE^16^. Similarly, we hypothesized that co-expression of animal MCU and EMRE proteins from chytrids would be necessary and sufficient to reconstitute uniporter activity. The phylogenetic distribution profile (presence/absence) across the MCU complex components revealed a strong co-evolution pattern, when only the true orthologous sequences were considered (Fig. 3a). Strikingly, we detected mt-Ca^2+^ uptake in yeast strains expressing animal MCUs from either *A. macrogynus* (Am-MCUa) or *S. punctatus* (Sp-MCU) with their respective EMREs (Am-EMRE1-2, Sp-EMRE) (Fig. 3b, c, Supplementary Fig. 3). Particularly, in *A. macrogynus* that encodes two MCU proteins, Am-MCUa and Am-MCUb, mt-Ca^2+^ uptake activity was detected only for the former, and showed different efficiency with its two encoded EMRE variants (Am-EMRE1 higher than Am-EMRE2). In contrast, we did not detect any mt-Ca^2+^ uptake in yeast strains expressing MCUP proteins from *A. macrogynus* (Am-MCUP1) and *S. punctatus* (Sp-MCUP), despite proper expression and localization (Supplementary Fig. 3, Supplementary Fig. 4). Similar results would be expected in other Holozoa despite the inability to detect EMRE by similarity searches. Indeed, the co-expression of MCU from the sea anemone *Nematostella vectensis* (Nv-MCU) with Hs-EMRE in yeast was able to reconstitute mt-Ca^2+^ uptake to a similar extent of a strain expressing Hs-MCU and Hs-EMRE (Supplementary Fig. 5). These results, together with the absence of MICU proteins in most fungal lineages, indicate that mt-Ca^2+^ uptake in fungal mitochondria, if it exists, is not mediated by MCUPs, or that a different —yet unknown—regulator is necessary. Instead, animal-like MCUs from chytrid fungi and Holozoa function similarly to the mammalian uniporter, in an EMRE-dependent fashion. Altogether, our evolutionary analyses and experimental results confirmed that MCU–EMRE interaction is conserved, and was already present in the last common ancestor of fungi and animals.

**Fig. 4:**
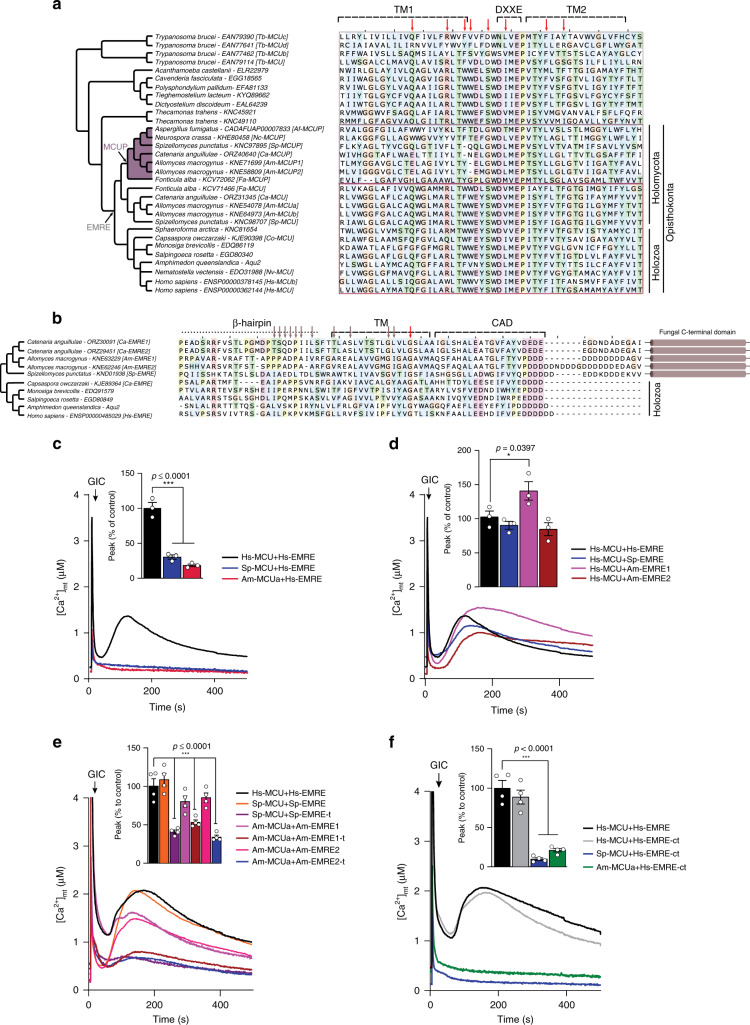
Evolution of MCU–EMRE interaction. **a**, **b** Phylogenetic trees of members of MCU (**a**) and EMRE (**b**) protein families, and sequence diversity of major domains. The sequence alignment of TM1 and TM2 of MCU sequences from 20 species is shown in **a**. The program Multi-Harmony^43^ was used to detect residues that are overall conserved but differ in the MCUP members (highest scoring positions are indicated with red arrows). The MCUP clade is shown in purple. The evolutionary point where the MCU proteins become EMRE dependent is shown in gray. The degree of conservation across the animal-related MCU members is very high in these loci, while few positions are Holomycota or Holozoa specific. Similarly, in **b** EMRE’s sequence diversity across opisthokonts is shown for the β-hairpin, the TM, and CAD domain. Residues found important for the interaction between MCU and EMRE in ref. ^20^ and fully conserved positions are indicated with gray and red arrows, respectively. **c**–**f** Representative traces and quantification of mt-Ca^2+^ transients in yeast cells expressing either human and animal-like *S. punctatus* and *A. macrogynus* MCU with human EMRE (*n* = 3) (**c**), species-specific EMRE with human MCU (*n* = 3) (**d**), animal-like *S. punctatus* and *A. macrogynus* MCU with their respective wild type or truncated (-t) EMREs (*n* = 4) (**e**) or with human EMRE fused to the chytrid extra C-terminal domain (*n* = 4) (**f**) upon glucose-induced calcium (GIC) stimulation in the presence of 1 mM CaCl_2_. All data represent mean ± SEM. *P* values are indicated in the different panels (**c**, **e**, **f** ***p < 0.001, one-way ANOVA with Dunnett’s multiple comparisons test; **d** *p = 0.012, one-way ANOVA with Dunnett’s multiple comparisons test). Source data for **c**–**f** are provided as a Source Data file.

Consistent with our hypothesis that MCUPs do not represent true functional orthologs of Hs-MCU, when comparing MCU sequences across eukaryotes we found that MCUPs lacked key residues conserved in the animal-like MCUs, despite retaining a DXXE motif (Fig. 4a, Supplementary Fig. 6a). Those residues have been previously shown to be important for MCU function and its interaction with EMRE^36^. Notably, animal and chytrid EMREs appeared highly divergent (Fig. 4b, Supplementary Fig. 6b), although the MCU interacting domain GXXXA/S/G and the polyaspartate tail necessary for the binding to MICU1 (ref. ^36^) were fully conserved. Interestingly, the fungal EMRE sequences contained an extra C-terminal domain that was not found in Holozoa, suggesting some degree of specialization. Thus, we hypothesized that Am-MCUa and Sp-MCU would have evolved to interact with EMRE proteins from the same or related species. To this goal, we measured mt-Ca^2+^ uptake in HeLa cells infected with lentiviral particles expressing either Hs-MCU or Am-MCUa and Sp-MCU (Supplementary Fig. 7a). Those proteins were expressed in addition to the endogenous Hs-MCU and Hs-EMRE. We found that mt-Ca^2+^ uptake in HeLa cells expressing either Am-MCUa or Sp-MCU was similar to uninfected control cells in response to histamine, differently from the overexpression of Hs-MCU, which resulted in a gain-of-function phenotype. We then measured mt-Ca^2+^ uptake in HeLa cells where MCU was stably knocked-down (sh-MCU) (Supplementary Fig. 7b). Here, we either re-introduced Hs-MCU or fungal MCU homologs (MCU and MCUP). As expected, the re-introduction of Hs-MCU resulted in a complete rescue of mt-Ca^2+^ uptake, which was similar to uninfected control cells (pLKO). Instead, unlike Hs-MCU, all the fungal MCUs and MCUPs were unable to rescue mt-Ca^2+^ uptake despite showing proper expression and insertion into the inner mitochondrial membrane (Supplementary Fig. 8), with a peak concentration of mt-Ca^2+^ similar to that of cells with MCU knock-down, due to the absence of a fungal EMRE. Furthermore, the expression of Am-MCUa and Sp-MCU in yeast mitochondria was unable to reconstitute mt-Ca^2+^ uptake in the presence of Hs-EMRE (Fig. 4c, Supplementary Fig. 9). Instead, Hs-MCU was functional when co-expressed with either Am-EMRE or Sp-EMRE (Fig. 4d, Supplementary Fig. 9). Altogether, these results suggested that the C-terminal domain of chytrid EMREs was dispensable for a functional interaction with Hs-MCU but necessary to activate animal-like fungal MCUs. Accordingly, we observed that the co-expression of Am-MCUa and Sp-MCU with Am-EMRE and Sp-EMRE lacking the extra C-terminal domain (EMRE-t) was unable to efficiently reconstitute mt-Ca^2+^ uptake (Fig. 4e, Supplementary Fig. 10a). However, while the fusion of Hs-EMRE with this extra chytrid C-terminal domain did not affect the function of Hs-MCU and thus of mt-Ca^2+^ uptake, it was not sufficient to reconstitute mt-Ca^2+^ uptake when co-expressed with Am-MCUa and Sp-MCU (Fig. 4f, Supplementary Fig. 10b). On the one hand, these findings hint at a possible activating role of the extra C-terminal domain of the fungal EMRE for the efficient regulation of animal-like MCUs function. On the other hand, they suggest that functional domains that are conserved between fungal and human EMREs are sufficient to regulate Hs-MCU activity.

## Discussion

Altogether, our identification of a strong co-evolution pattern between MCU, MICU, and EMRE provides an explanation to an elusive evolutionary paradox: the presence of uniporter homologs in species with MCU but no MICU1 homologs and no detectable mt-Ca^2+^ uptake^25,26^. We demonstrate that an animal-related MCU complex has been lost early within the evolution of most fungal clades, which retained only fungal-specific paralogous MCU proteins (MCUPs). These MCUPs, which are paralogous to Hs-MCU, have been so far wrongly considered functionally equivalent to Hs-MCU and deeply studied for this reason. This is the case, for instance, of the Ascomycota MCUP proteins (e.g., *N. crassa*) that have been recently used as models for understanding structure and regulation of the human uniporter channel^20–24^. Consistently, we and others find that fungal MCUPs are unable to reconstitute or rescue mt-Ca^2+^ uptake in yeast or HeLa cells lacking MCU, respectively^17,24,29^. Similarly, the same fungal MCUP sequences used for investigating structure and function of the mammalian uniporter^21,22,24^ failed to mediate mt-Ca^2+^ uptake when expressed in yeast mitochondria or in MCU knockout HeLa cells (Supplementary Fig. 11). Instead, we show that only fungal species having both MCU and EMRE sequences (animal-like MCUs), such as *A. macrogynus* and *S. punctatus*, are able to mediate mt-Ca^2+^ uptake. Those observations together with the significant structural and sequence differences found between MCUs and MCUPs^20^ question whether fungal MCUPs function as classical Ca^2+^ uniporters.

The remarkable presence/absence pattern across eukaryotes of MCU and MICU—when only the orthologs of the two protein families are considered—indicates strong co-evolution and functional interdependence. Indeed, MICU1 plays a key role in regulating the gating and activation properties of MCU^9^. Thus, the evolutionary coupling of uniporter’s gating mechanism with its regulator suggests a dependency on MICU for Ca^2+^ homeostasis and cell physiology. Consistently, across the 1156 species in our analysis, MCU and MICU co-occur in 1144 (~99%), whereas only 12 species have one of the two components. Most such incongruencies are likely to result from assembly, annotation, or identification errors. In fact, we expect MICU to be encoded in all species with a functional MCU complex, with the only likely exception being Onchocercidae, a family of parasitic nematodes. In our dataset all three species in the clade (*Brugia malayi*, *Onchocerca volvulus*, and *Loa loa*) appear to lack MICU1 homologs in the presence of MCU (Supplementary Fig. 1), raising the possibility that MICU is indeed lost in the clade rather than being undetected. If this preliminary observation is confirmed, this would make these parasites interesting targets to explore further the physiological significance of MICU1.

**Fig. 5:**
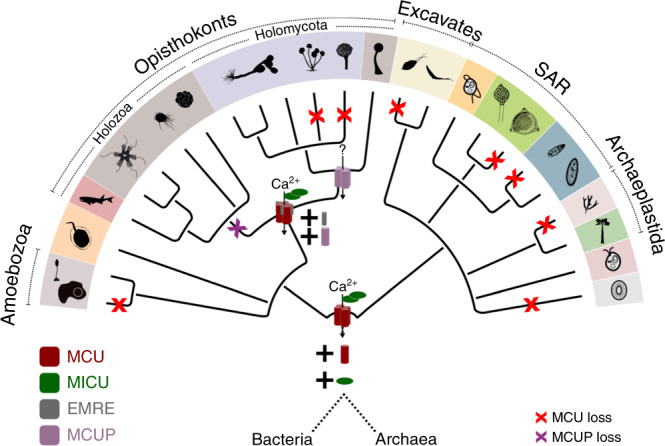
Schematic representation of the evolution of the MCU complex in eukaryotes. Changes in the composition of the uniporter complex are shown in the respective taxonomic levels. The MCU–MICU complex emerges at the origin of eukaryotes, followed by the addition of EMRE in opisthokonts. MCUP is only found in Holomycota. Losses of MCU in the various eukaryotic lineages are indicated in red and the loss of MCUP in Holozoa in purple. The color code is the same as in Fig. 1, and the taxonomic tree structure and silhouette images of the different taxonomic groups are as in Fig. 3a.

Our phylogenomic analysis identifies non-metazoan EMRE sequences and demonstrates the ancestrally essential role of EMRE in mt-Ca^2+^ homeostasis. Our results imply that EMRE, previously thought to be an animal-specific innovation^15,16,27^, formed part of an animal-like machinery in the common ancestor of opisthokonts, and was lost secondarily in the evolution of fungi, together with the other components of the animal-like mt-Ca^2+^ uptake machinery (Fig. 5). These results have major implications for structural and functional studies of the uniporter. Indeed, members of the orthologous MCU complex in basal fungi constitute relevant targets for future research and comparative structural analyses, particularly for identifying key MCU–EMRE interactions. Our results show that human MCU can function in the presence of both human and chytrid EMREs, whereas chytrid MCUs such as Am-MCUa and Sp-MCU can only reconstitute mt-Ca^2+^ uptake when co-expressed with their corresponding chytrid EMREs. These findings indicate a tight co-evolution between MCU and EMRE proteins, which we know to functionally and physically interact, and provide the framework to understand the sequence determinants of this interaction. Finally, our work underscores that accurate phylogenomic analyses can resolve apparent evolutionary riddles while making explicit functional predictions that can drive future experiments.

## Methods

### Sequence data and homology searches

The protein sequences encoded in 1156 completely sequenced eukaryotic genomes were retrieved from Ensembl DB v91, and v37 of Ensembl Metazoa, Plants, Fungi, and Protists (see Supplementary Data 1). Only the genome of Catenaria anguillulae PL171 was added from Ensembl Fungi v41. For each of the protein families studied, homologs were selected on the basis of sequence similarity and phylogenetic analysis. HMMER searches were performed using HMMER 3.1b2 (ref. ^37^) and using the Gathering Cut-Off threshold (–cut_ga) when the raw HMM profile from Pfam was used, an *e*-value threshold of 10^−2^ otherwise. For all BLAST searches low-complexity regions in the query sequence (default parameter) were filtered out to minimize the number of false positives and an *E*-value threshold of 10^−5^ was used. Conserved domains in all retrieved sequences were annotated using the HMM profiles of Pfam release 30.0. For MCU, proteins in our database containing at least one MCU (Pfam: PF04678 [https://pfam.xfam.org/family/PF04678]) domain were detected using an HMMsearch. In total, 1076 protein sequences were selected for subsequent analysis. For MICU, 2105 protein sequences were retrieved from a BLAST search using Hs-MICU1 (Uniprot: Q9BPX6 [https://www.uniprot.org/uniprot/Q9BPX6]) as a query. HMMscan was used to search for additional domains in the retrieved sequences using all the Pfam domain profiles, and all the sequences with detected at least one Mito_carr domain (Pfam: PF00153 [https://pfam.xfam.org/family/PF00153]) were classified as the members of the mitochondrial carrier family (slc25a12-Aralar homologs). The Aralar-related sequences (Mito_carr domain containing) were clustered together clearly as a monophyletic clade in a phylogenetic tree, in the exclusion of known MICU sequences, and were excluded. The remaining 651 MICU sequences were re-aligned and a new phylogenetic tree with the same methods was reconstructed. EMRE sequences are characterized by a DDDD (Pfam: PF10161 [https://pfam.xfam.org/family/PF10161]) domain. Their short length and low sequence conservation makes detection strikingly difficult, which explains why in some cases EMRE appears to be missing even from some animal genomes. HMMER searches with the “gathering” (–cut_ga) threshold were performed, and all detected homologs were retrieved and re-aligned, for a new HMM profile to be built and used to search back the genome database in a second iteration. One EMRE sequence was detected in *A.macrogynus* in the first search, while one more sequence in the same species, as well as in *S. punctatus* and *C. anguillulae*, were detected in the second iteration. The detected EMRE sequences in this second iteration are those that were further considered for all analyses. For NCLX (NCKX6), the Na_Ca_ex Pfam domain was initially used to retrieve protein sequences. However, this domain characterizes a ubiquitous superfamily of sodium/calcium exchangers that regulate intracellular Ca^2+^ concentrations in many cell types. Therefore, selection of NCKX6 (NCLX) homologs based on the Na_Ca_ex Pfam domain using HMMER returned 4024 hits. To narrow down the number of hits for more accurate alignment and phylogenetic reconstruction, we used the human NCLX sequence as a query (uniprot: Q6J4K2 [https://www.uniprot.org/uniprot/Q6J4K2]) for a blast search, retrieving 2105 sequences for phylogenetic analysis. Using the human members as reference, 1391 sequences across eukaryotes were selected as related to the NCLX clade (NCLX orthologs).

### Phylogenetic analysis

Sequences of all the protein families described were aligned, and the alignment was trimmed and used to compute a phylogenetic tree. The selected homologous proteins were aligned with MAFFT v7.394 (ref. ^38^) (E-INS-I for MICU, EMRE, and NCLX families and L-INS-i for the MCU family, based on their multi/single-domain architecture) and a soft trimming was applied, filtering out positions in the alignment with gaps in more than 99% of the sequences using trimAl^39^ (−gt 0.01). IQ-TREE v1.6.8 (ref. ^40^) was used to derive Maximum Likelihood (ML) trees. LG was selected as the base model to test all rate-heterogeneous models using the “-mset LG” parameter (26 protein models, combinations of invariable sites +I, discrete Gamma model with 4 rate categories +G, the FreeRate model +R with 2–10 categories, and empirical AA frequencies estimated by the data). The best-fit models, chosen according to the Bayesian information criterion (BIC), were LG + R10 for the MCU and MICU alignments and LG + F + R10 for NCLX alignment. Branch supports were obtained using the ultrafast bootstrap implemented in the IQ-TREE program with 1000 replicates. The ETE Toolkit^41^ was used for all taxonomic and phylogenetic tree operations and visualization. Sequence alignments were visualized using Jalview2 (ref. ^42^), and the Multi-Harmony^43^ method was used identify patterns of variation across the different protein clades, positions conserved across the animal MCUs and the animal-like fungal MCUs but not in the MCUPs. Raw phylogenetic trees in newick format can be found in Supplementary Data 2.

### Cell lines

HeLa cells stably expressing a wild-type mitochondrial matrix-targeted GFP-aequorin (mt-AEQ) were generated as in ref. ^18^. Mt-AEQ HeLa cells stably overexpressing Hs-MCU, Sp-MCUP, Sp-MCU, Am-MCUP1, Am-MCUa, Am-MCUb, Af-MCUP, and Nc-MCUP from the pLX304 lentiviral vector were then generated by viral transduction according to the manufacturer’s instructions. MCU knock-down mt-AEQ HeLa cells were generated by viral transduction using a pLKO vector expressing an shRNA targeting Hs-MCU (sh-MCU; Sigma-Aldrich, TRCN0000133861). Control cells were generated using the empty vector pLKO (Addgene #8453). Mt-AEQ MCU knockout (MCU-KO) HeLa cells transiently expressing Hs-MCU, Ce-MCUP, Fg-MCUP, Ma-MCUP, and Nf-MCUP from the pLX304 lentiviral vector were generated by transfection using the X-tremeGene HP DNA transfection reagent (Roche), according to the manufacturer’s instructions. cDNAs for Sp-MCUP, Sp-MCU, Am-MCUP1, Am-MCUa, Am-MCUb, Ce-MCUP, Fg-MCUP, Ma-MCUP, and Nf-MCUP expression without a stop codon were codon optimized for human expression, synthesized de novo in the PuC57 vector, and amplified with flanked attB1 and attB2 sites by PCR (see Supplementary Table 1). PCR products were first integrated into the pDONR221 vector and then into the pLX304 destination vector by site-specific recombination according to the manufacturer’s instructions (Life Technologies). All cell lines were grown in high-glucose Dulbecco’s modified Eagle’s medium supplemented with 10% FBS at 37 °C and 5% CO_2_.

### Yeast strains

A yeast strain expressing mt-AEQ with Hs-MCU and Hs-EMRE was generated as in refs. ^17^^,^^18^. To generate yeast strains co-expressing mt-AEQ together with different combinations of fungal and human MCU and EMRE orthologs, cDNAs were amplified from the pLX304 vector (see Supplementary Table 2) and cloned into the yeast expression plasmids p423GPD (Hs-MCU, Sp-MCUP, Sp-MCU, Am-MCUP1, Am-MCUa, Am-MCUb, Ce-MCUP, Fg-MCUP, Ma-MCUP, and Nf-MCUP) and p425GPD (Hs-EMRE, wild-type and C-terminal domain truncated Sp-EMRE, Am-EMRE1, and Am-EMRE2, and Hs-EMRE with an addition of chytrid C-terminal domain from Sp-EMRE). The YPH499 strain was then transformed with the respective plasmids and transformants were selected on synthetic dextrose plates, with adenine, lysine, and tryptophan as selection markers.

### MCU knockout in HeLa cells

Mt-AEQ HeLa cells with knockout of MCU were generated by CRISPR targeting^44^. In brief, a cDNA encoding NLS-Cas9 was isolated from pX330 (Addgene #42230) by *Eco*RI digestion and cloned upstream of the ubiquitous CAG promoter. Two sets of single-guide RNA (sgRNA) were designed using an online tool CRISPOR (MCU-KO1 gRNAa: 5′-CACCGCAGGAGCGATCTACCTGCGG-3′; MCU-KO2 gRNAa: 5′-CACCGTGAACTGACAGCGTTCACGC-3′). For each sgRNA, complementary oligonucleotides containing the target sgRNA sequences were annealed and cloned into the *Bbs*I site of the pX330-Puro-ccdB vector^45^. After which, pX330-Puro-ccdB vector containing sgRNAs were transfected into mt-AEQ HeLa cells using the Lipofectamine 3000 Reagent (Life Technologies), according to the manufacturer′s instructions and selected with 2 μg/ml puromycin for 2 days. Cells were then seeded at a density of 1 cell per well and expanded. Gene knockout was screened by PCR (MCU-KO Forward1: 5′-GCGTGTAGTTGAGAGTTACAGC-3′; MCU-KO Forward2: 5′-TTTTATAAGCCAGTTCCCAGAATAACCT-3′; MCU-KO Reverse: 5′-GTTCATCCTTGCTCATGGCATT-3′) and confirmed by sequencing and western blot using the following antibodies: anti-MCU (Sigma-Aldrich, HPA01648, 1:1000), anti-EMRE (Santa Cruz Biotechnology, sc-86337, 1:1000), anti-ACTIN (Sigma-Aldrich, A2228, 1:5000).

### Isolation of crude mitochondria from HeLa cells

Crude mitochondria were isolated from HeLa cells as described^17^. Briefly, HeLa cells were grown to confluency, rinsed with PBS, and resuspended in ice-cold isolation buffer (IB: 220 mM mannitol, 70 mM sucrose, 5 mM HEPES-KOH pH 7.4, 1 mM EGTA-KOH pH 7.4, protease inhibitors). Cells were permeabilized by nitrogen cavitation at 600 psi for 10 min at 4 °C and then centrifuged at 600 × *g* for 10 min. The supernatant was transferred into new tubes and centrifuged at 8000 × *g* for 10 min at 4 °C. The resulting pellet containing crude mitochondria was resuspended in IB for protein topology analysis.

### Analysis of mitochondrial protein topology

Proteinase K (PK) protection assay was performed on mitochondria isolated from HeLa cells^17^. Roughly, 30 µg of freshly isolated mitochondria were gently resuspended in 30 µl of IB buffer with either increasing concentrations of digitonin or 1% Triton X-100 in the presence of 100 µg/ml PK and incubated at room temperature for 15 min. The reaction was stopped by the addition of 5 mM PMSF, followed by incubation on ice for 10 min. Samples were mixed with 10 µl of 4× Laemmli buffer containing 10% 2-mercaptoethanol and boiled for 5 min at 98 °C for immunoblot analysis. Immunoblotting was performed according to the standard procedures using the following antibodies: anti-V5 (Life Technologies, R96025, 1:5000); anti-TIM23 (BD Bioscience, 611222, 1:5000); anti-TOM20 (Abcam, ab56783, 1:1000); anti-Cyclophilin D (Cyp D) (Abcam, ab110324, 1:1000). TOM20, TIM23, and Cyp D were used as controls for integral mitochondrial outer membrane, inner membrane, and soluble matrix-targeted proteins, respectively.

### Subcellular fractionation of yeast cells

Expression and subcellular localization of heterologous expressed proteins in yeast was tested by immunoblot analysis of cytosolic and mitochondrial fractions isolated from recombinant yeast strains^17^. Briefly, yeast cells were grown at 30 °C in a selective lactate medium supplemented with the respective selection markers till an OD ~0.8. The cell pellet was resuspended in a buffer containing buffer 0.6 M sorbitol, 20 mM HEPES/KOH pH 7.2, 80 mM KCl, and 1 mM PMSF, and vortexed five times for 30 s with glass beads (425–600 μm diameter), with a 30 s cooling interval in between to break cell wall and plasma membrane. After the first centrifugation step at 1000 × *g* for 5 min at 4 °C, the supernatant was further centrifuged at 20,000 × *g* for 10 min at 4 °C to obtain the mitochondrial fraction (pellet). The supernatant (cytosolic fraction) was precipitated with trichloroacetic acid at −20 °C for 1 h, washed once with cold acetone, and centrifuged at 20,000 × *g* for 10 min at 4 °C. Both cytosolic and mitochondrial fractions were analyzed with immunoblotting according to the standard procedures using the following antibodies: anti-MCU (Sigma-Aldrich, HPA016480, 1:1000); anti-EMRE (Santa Cruz Biotechnology, sc-86337, 1:1000); anti-V5 (Life Technologies, R96025, 1:5000); anti-AEQ (Merck/Millipore, MAB4405, 1:1000); anti-YME1; anti-PGK1 (Life Technologies, 459250, 1:10,000).

### Measurements of mitochondrial calcium uptake in yeast and HeLa cells

In vivo analyses of mitochondrial Ca^2+^ uptake in intact yeast and HeLa cells were performed using an aequorin-based measurement^46^. Aequorin is widely used as a genetically encoded Ca^2+^ indicator (GECI) for measurements of mt-Ca^2+^ kinetics because it offers several advantages. Besides being targeted with precision to the organelle, it functions over a wide range of Ca^2+^ concentrations, and shows low buffering capacity, despite achieving a limited spatial resolution compared to fluorescent GECIs^46^. Briefly, yeast cells were collected at an OD ~0.8, washed three times with milliQ water, and starved for 1.5 h at room temperature in a nutrient-free buffer (NFB, 100 mM Tris, pH 6.5 (1 × 10^8^ cells per ml). Afterwards, cells were collected at 3500 r.p.m. for 5 min and resuspended in NFB to a higher density (25 × 10^8^ cells per ml) in the presence of 50 μM native coelenterazine (Abcam, ab145165) to reconstitute the photoprotein aequorin. After 30 min in the dark at room temperature, 0.5 × 10^8^ cells per well were plated into a white 96-well plate and Ca^2+^-dependent light kinetics was recorded upon stimulation with 1 mM CaCl_2_ and 100 mM glucose, at 469 nm every 0.5 s interval in a MicroBeta2 LumiJET Microplate Counter. At the end of each experiment, cells were lysed with 1 mM digitonin for 5 min at 37 °C and any residual aequorin counts were collected upon the addition of CaCl_2_ to a final concentration of 140 mM. For mt-Ca^2+^ uptake measurement in mt-AEQ HeLa cells, those were seeded at 25,000 cells/well of white 96-well plates in growth medium overnight. Afterwards, mt-AEQ was reconstituted with 2 μM native coelenterazine for 2 h at 37 °C and Ca^2+^-dependent light kinetics were recorded upon 100 μM histamine stimulation at 469 nm every 0.1 s interval. At the end of each experiment, cells were lysed with a solution containing 0.5% Triton X-100 and 10 mM CaCl_2_ to record the released residual aequorin counts.

### Quantification of calcium transients

Quantification of mt-Ca^2+^ concentration was performed using a MATLAB software as in ref. ^18^. The dynamics of mt-Ca^2+^-dependent luminescence signal was smoothed by the cubic spline function:

1 $$p\mathop {\sum}\limits_1^n {\left( {y_i - f(x_i)} \right)^2 + (1 - p)f\left( {\frac{{{\mathrm{d}}^2f}}{{{\mathrm{d}}x^2}}} \right)^2{\mathrm{{d}}}x},$$

where *p* is a smoothing parameter, controlling the tradeoff between fidelity to the data and roughness of the function estimate, *f* is the estimated cubic spline function to minimize the above function, and *x*_*i*_ and *y*_*i*_ are the dynamical data points. Here, *p* is set at 0.5. Parametrization of the Ca^2+^-dependent luminescence kinetics was performed in order to determine the maximal amplitude of the luminescence signal (peak) and the left slope of the bell-shaped kinetic trace. Aequorin-based luminescence signal calibration into mt-Ca^2+^ concentration was performed using the algorithm reported in ref. ^46^ for wild-type aequorin and native coelenterazine, with the following formula:

2 $$[{\mathrm{{Ca}}}^{2 + }](M) = \frac{{\left( {\frac{L}{{L_{{\it{\max}}}}} \times \lambda } \right)^{\frac{1}{n}} + \left( {(\frac{L}{{L_{{\it{\max}}}}} \times \lambda )^{\frac{1}{n}} \times K_{TR}} \right) - 1}}{{K_R - \left( {(\frac{L}{{L_{{{\max}}}}} \times \lambda )^{\frac{1}{n}} \times K_R} \right)}},$$

where *λ* = 1, *K*_*R*_ = 7.23 × 10^6^, *K*_*TR*_ = 120, and *n* = 2.99 are the calibration values used for WT aequorin and native coelenterazine.

### Experimental data analysis

Data are represented as mean ± SEM and the statistical analysis of each experiment is described in the figure legends including the statistical tests used and the exact value of biological replicates. For each biological replicate experiment at least three technical replicates were used for quantification and data analysis. Normal distribution was tested by Shapiro–Wilk normality test. Statistical tests between multiple datasets and conditions were carried out using one-way analysis of variance followed by Dunnett’s multiple comparison tests. Statistical analyses were performed using GraphPad Prism (GraphPad Software, version 8).

### Reporting summary

Further information on research design is available in the Nature Research Reporting Summary linked to this article.

## Supplementary information

Supplementary Information

Peer Review

Reporting Summary

Description of Additional Supplementary Files

Supplementary Data 1

Supplementary Data 2

## Data Availability

The data that support the findings of this study are available from the corresponding authors upon reasonable request. All genome data and predicted peptide sets are publicly available and were downloaded from Ensembl-v91 (https://www.ensembl.org/), and Ensembl fungi (https://fungi.ensembl.org/), metazoa (https://metazoa.ensembl.org/), plants (https://plants.ensembl.org/) and protists (https://protists.ensembl.org/index.html) v37. The raw HMM profiles of the different protein families were downloaded from Pfam release 30.0 (https://pfam.xfam.org/). Hs-MICU1 (MICU1_HUMAN) and NCLX (NCLX_HUMAN) sequences were downloaded from the Uniprot database (https://www.uniprot.org/uniprot/Q9BPX6 and https://www.uniprot.org/uniprot/Q6J4K2). The source data underlying Figs. 3b, c, 4c–f, and Supplementary Figs 3, 4, 5, 7, 8, 9, 10, and 11 are provided as a Source Data file. Full scans of the blots are available in Supplementary Fig. 12. All genomic data sources and versions analyzed in the study and the raw phylogenetic trees in newick format are provided in Supplementary Data 2. The silhouette images used in Figs. 3 and 5 were downloaded from PhyloPic (http://phylopic.org/) or wikipedia (https://www.wikipedia.org/), or designed by the A.P. in Inkscape (https://inkscape.org/).

## Source data

Source Data

## Code availability

There is no specific new code developed for this project; custom python scripts and different programs were used as described in the Methods. These are available with no restriction under request.
