# Supplementary material for: Discovery of EMRE in fungi resolves the true evolutionary history of the mitochondrial calcium uniporter
Source: Nat Commun. 2020 Aug 12;11:4031. doi: 10.1038/s41467-020-17705-4 (PMC7423614; doi:10.1038/s41467-020-17705-4)
Supplement: Supplementary file 1 — Supplementary Information [file 41467_2020_17705_MOESM1_ESM.pdf]

Supplementary Information

Discovery of EMRE in fungi resolves the true evolutionary history of the mitochondrial calcium uniporter

Pittis et al.

Supplementary Figures 1-12
Supplementary Tables 1-2

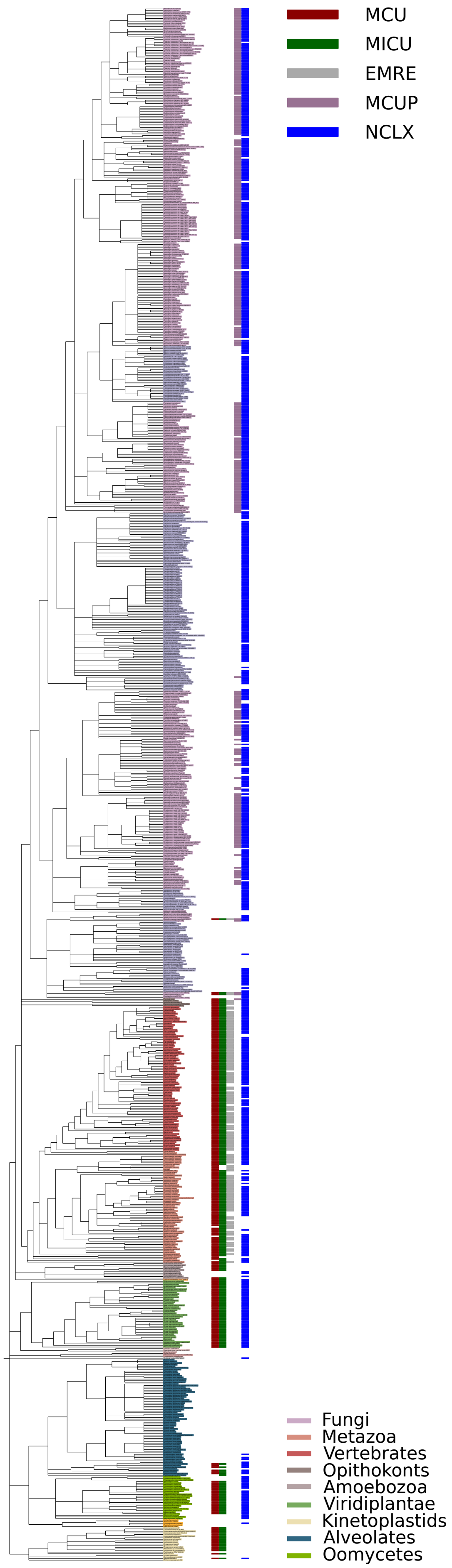

Supplementary Fig. 1. Phylogenetic distribution of the mitochondrial calcium transporter complex protein families. The tree is collapsed to the species level, resulting in 969 species. Extended version of Figure 1, including also the distribution of NCLX, which appears to be largely uncoupled to that of the MCU complex members.

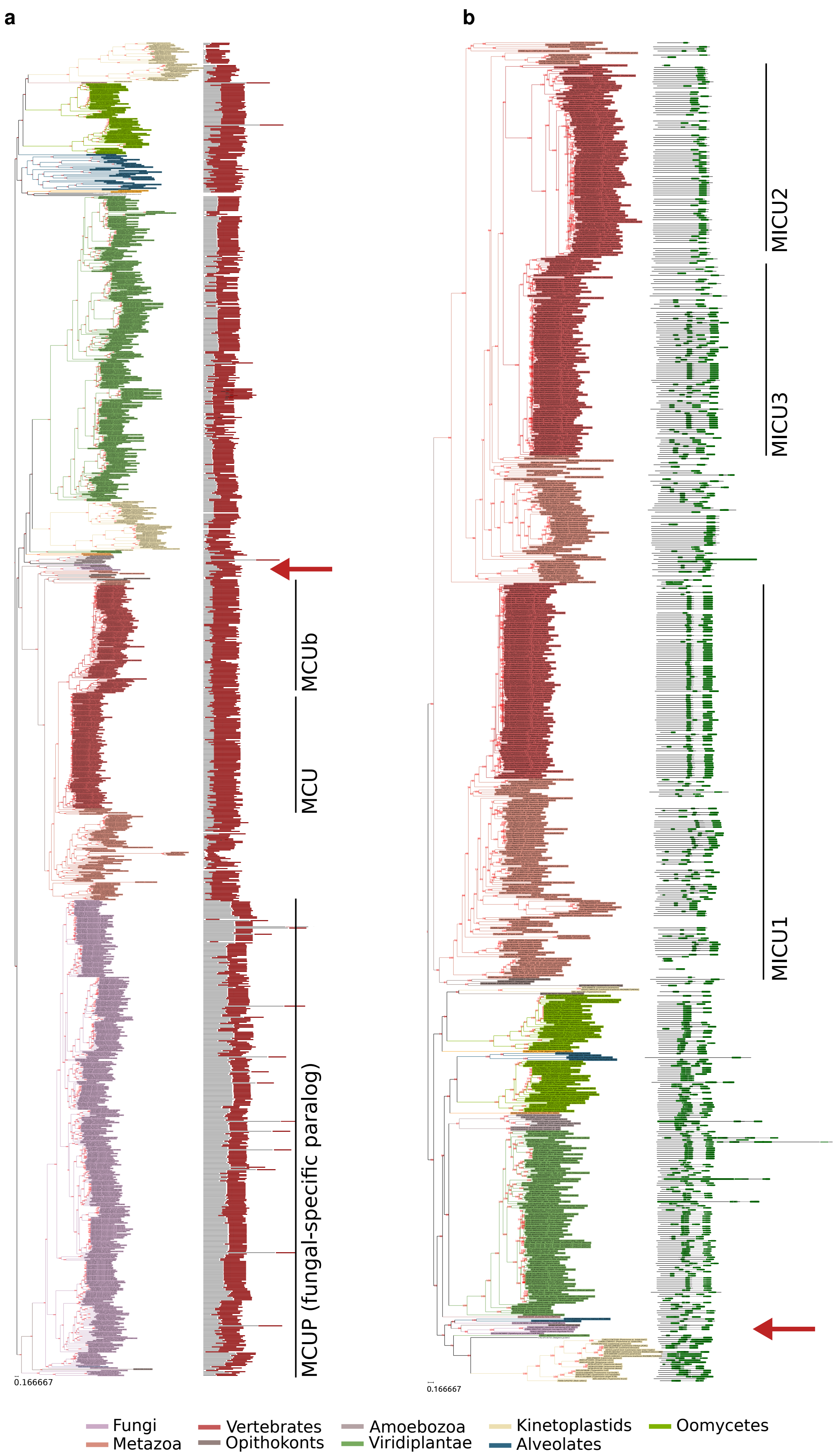

Supplementary Fig. 2. Maximum likelihood (ML) phylogenetic trees of MCU and MICU families. Phylogenies of the 1,064 MCU sequences (a) and the 651 MICU sequences (b). Full version with sequences ids of Fig. 2a,b. The UFBoot (Ultrafast Bootstrap Approximation) support values, as implemented in IQ-TREE 1.6.8, are indicated in red. The main subfamilies are shown, based on the human representatives. The basic domain architecture according to Pfam is plotted on the right. The MCU family is characterized by the presence of one “MCU” domain (in red), while the typical MICU sequence consists of two “EF-hand” domains (in green). In both (a) and (b) position of the animal related MCU and MICU sequences from fungi and F.alba are indicated with a red arrow. See also Methods.

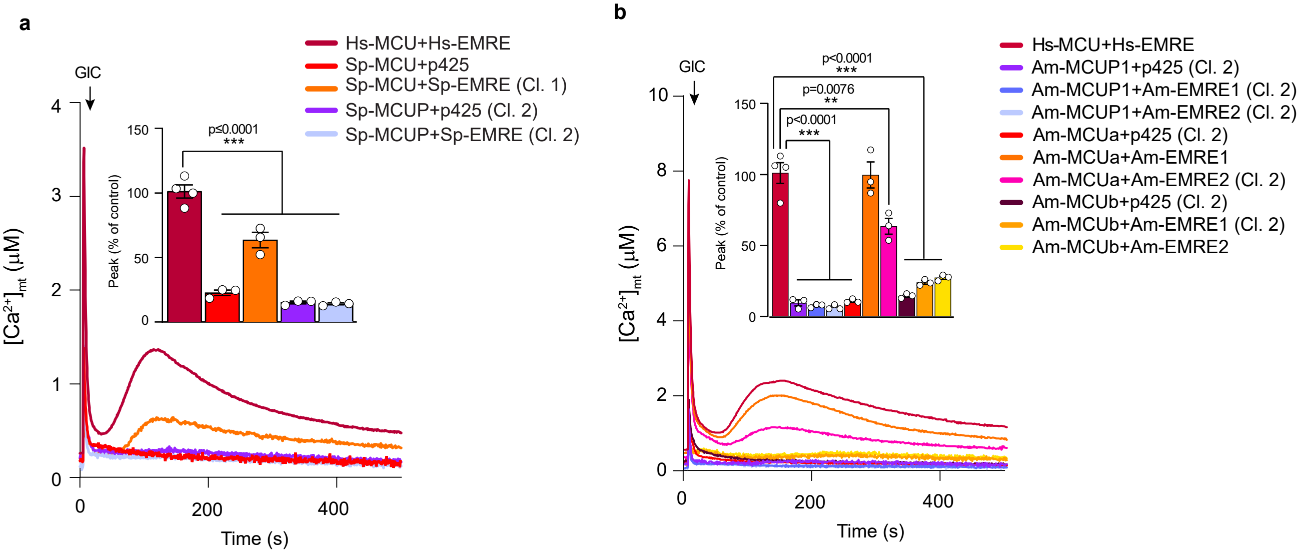

Supplementary Fig. 3. Reconstitution of mt-Ca^{2+} uptake in yeast cells expressing *S. punctatus* or *A. macrogynus* MCU and EMRE homologs. a,**b**, Representative traces and quantification of mt-Ca^{2+} transients in yeast cells expressing human MCU (Hs-MCU) (n=4) or MCU orthologs from *S. punctatus* (Sp-MCU, Sp-MCUP) (n=3) (**a**) and *A. macrogynus* (Am-MCUa, Am-MCUb, Am-MCUP1) (n=3) (**b**) with either their respective EMRE orthologs (Hs-EMRE, Sp-EMRE, Am-EMRE1, Am-EMRE2) or an empty vector (p425) upon glucose-induced calcium (GIC) stimulation in presence of 1 mM $CaCl_2$. All data represent mean ± SEM. *P* values are indicated in the different panels (**a-b**: ***p < 0.001, one-way ANOVA with Dunnett's Multiple Comparisons Test). Source data are provided as a Source Data file.

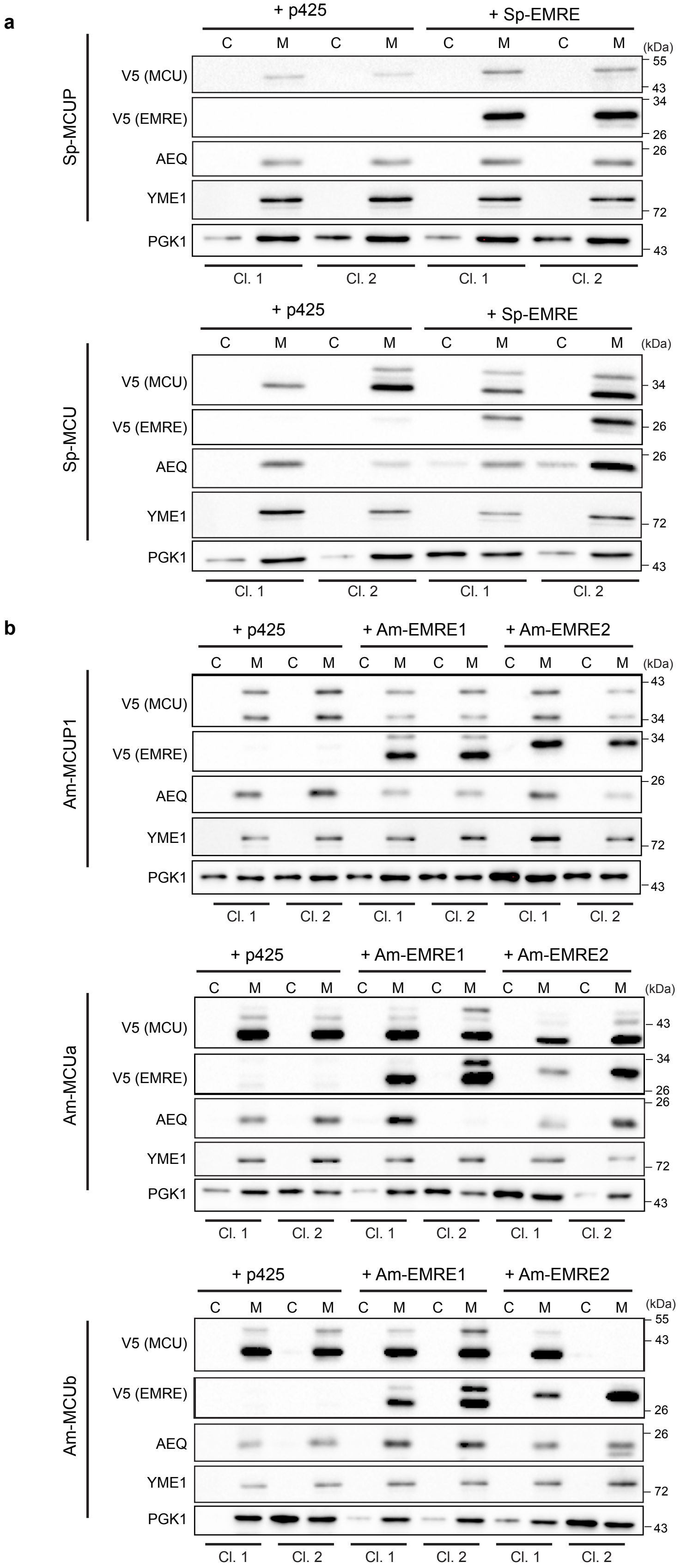

Supplementary Fig. 4. Heterologous expression of *S. punctatus* and *A. macrogynus* MCU and EMRE homologs in yeast. a,**b**, Immunoblot analysis of cytosolic (C) and mitochondrial (M) fractions isolated from yeast clones (Cl.) expressing mt-AEQ together with either (**a**) *S. punctatus* MCU (Sp-MCUP, Sp-MCU) and EMRE (Sp-EMRE) homologs or(**b**) *A. macrogynus* MCU (Am-MCUP1, Am-MCUa, Am-MCUb) and EMRE (Am-EMRE1, Am-EMRE2) homologs fused to a C-terminal V5-tag, using the following antibodies: α-V5 (Life Technologies, R96025), α-AEQ (Merck/Millipore, MAB4405), α-YME1, PGK1 (Life Technologies, 459250). YME1 was used as control for yeast mitochondrial targeted protein and PGK1 was used as control for cytosolic protein. Source data are provided as a Source Data file.

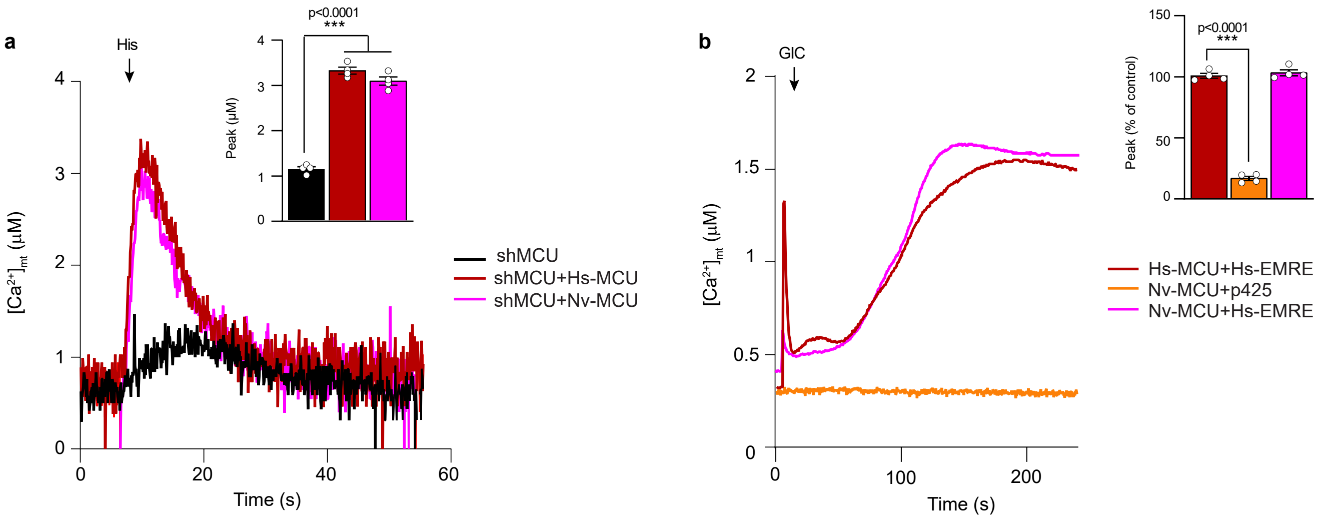

Supplementary Fig. 5. Reconstitution of mt-Ca^{2+} uptake in HeLa and yeast cells expressing *N. vectensis* MCU. **a**, Representative traces and quantification of mt-Ca^{2+} transients in MCU knockdown (shMCU) HeLa cells expressing human (Hs-MCU) and *N. vectensis* MCU (Nv-MCU) upon histamine (His) stimulation (n=4). **b**, Representative traces and quantification of mt-Ca^{2+} transients in yeast cells expressing Nv-MCU with either empty vector (p425) or human EMRE (Hs-EMRE) upon glucose-induced calcium (GIC) stimulation in presence of 1 mM $CaCl_2$ (n=4). All data represent mean ± SEM. *P* values are indicated in the different panels (**a-b**: ***p < 0.0001, one-way ANOVA with Dunnett's Multiple Comparisons Test). Source data are provided as a Source Data file.

a

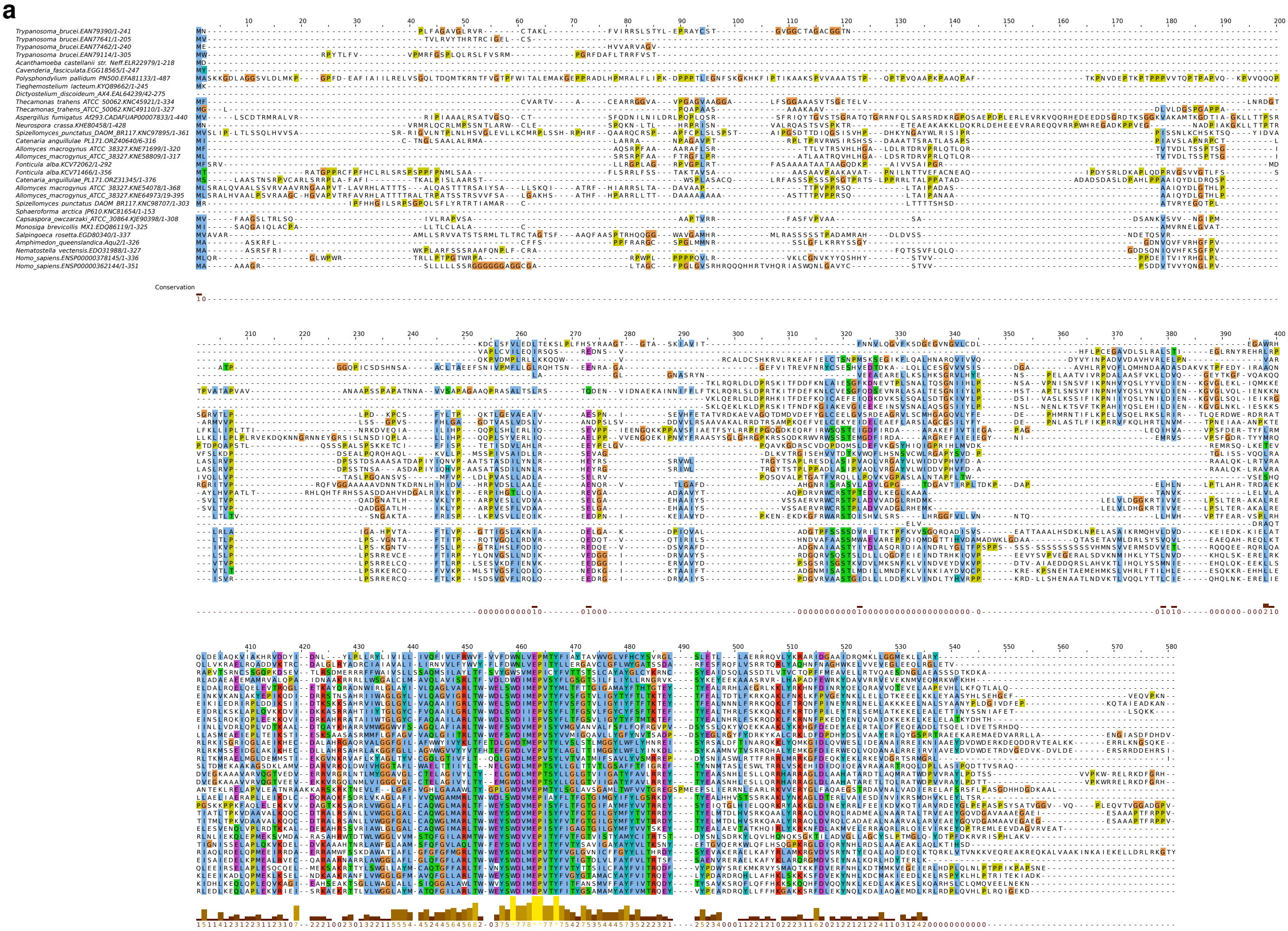

b

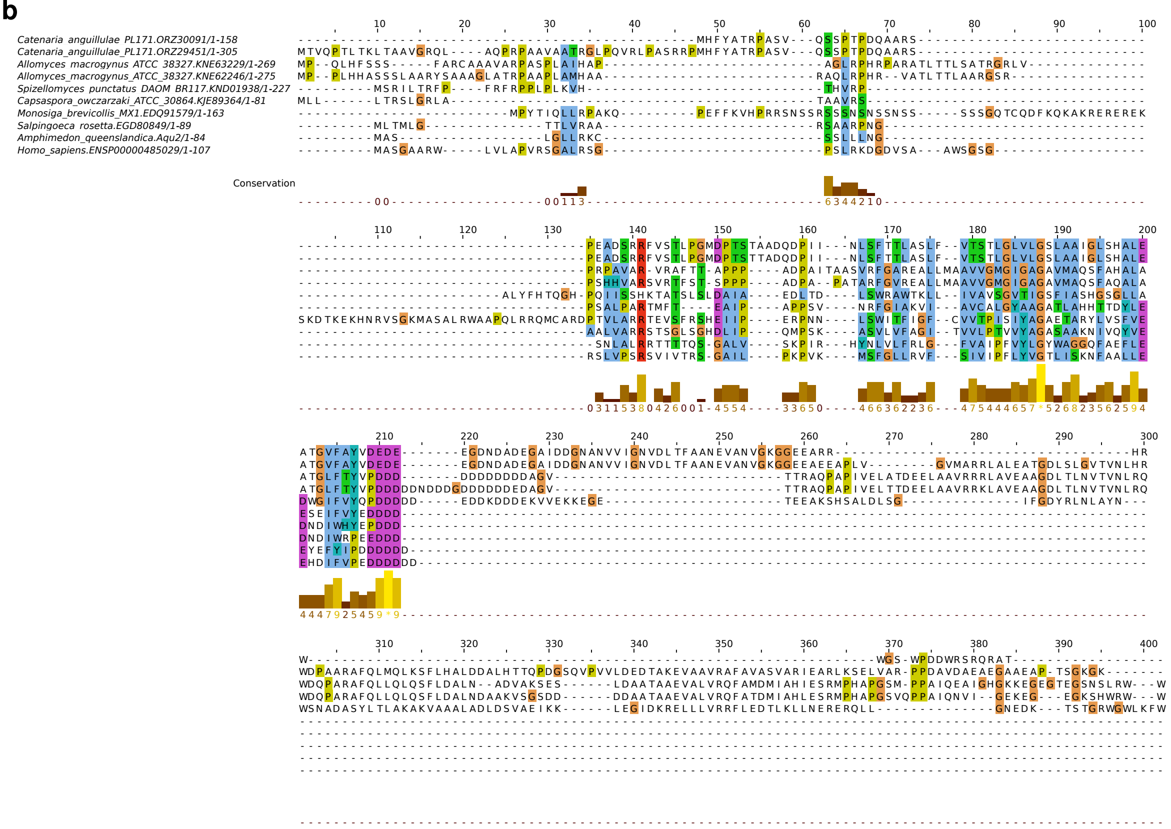

Supplementary Fig. 6. a,b, Multiple sequence alignments of opisthokont members of MCU (a) and EMRE (b) families. In (a) MCU regions poorly aligned or specific to non-opisthokont species have been removed, whereas in (b) the full EMRE sequences are shown, for all the species in (a) where EMRE was detected.

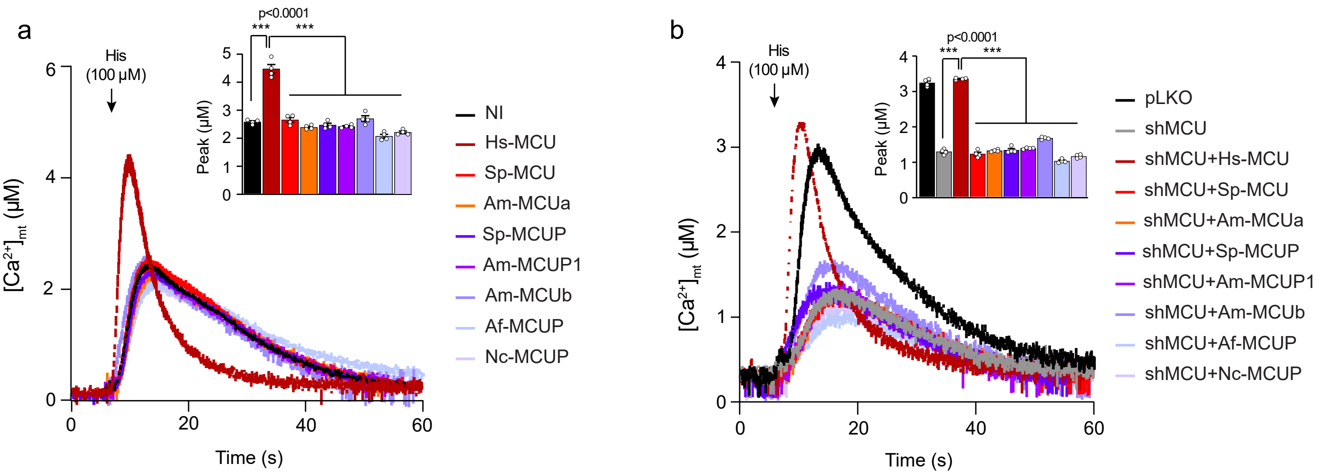

Supplementary Fig. 7. Reconstitution of mt-Ca²⁺ uptake in HeLa cells expressing fungal MCU homologs. a,**b**, Quantification of mt-Ca^{2+} transients in either wild-type (a) or wild-type (pLKO) and MCU knockdown (shMCU) (b) HeLa cells expressing human (Hs-MCU) or different fungal MCU homologs from *S. punctatus* (Sp-MCU, Sp-MCUP), *A. macrogynus* (Am-MCUa, Am-MCUb, Am-MCUP1), *A. fumigatus* (Af-MCUP) and *N. crassa* (Nc-MCUP) upon histamine (His) stimulation (n=4). NI, not infected. All data represent mean ± SEM. *P* values are indicated in the different panels (**a-b**: ***p < 0.0001, one-way ANOVA with Dunnett's Multiple Comparisons Test). Source data are provided as a Source Data file.

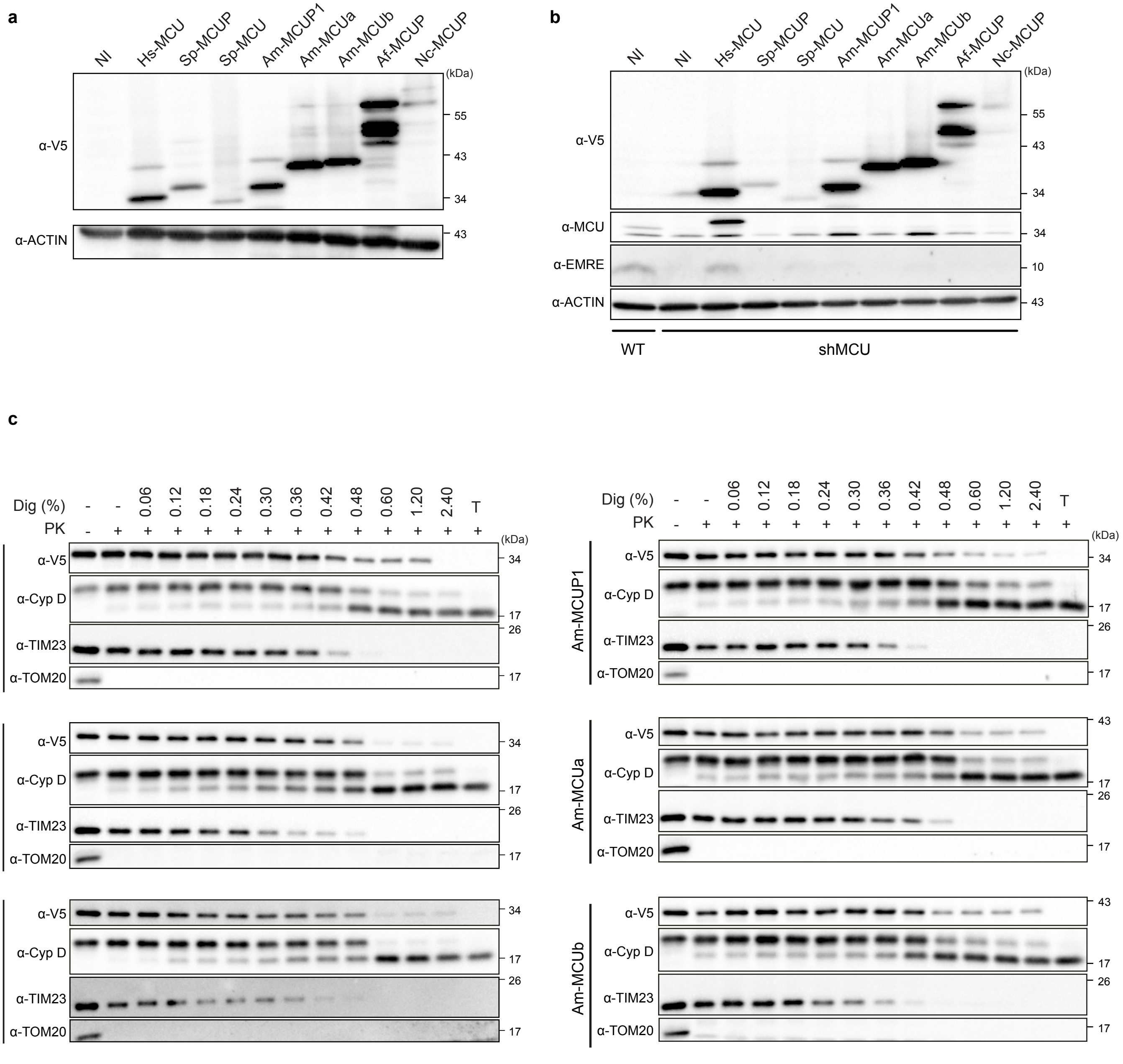

Supplementary Fig. 8. **Expression and localization of fungal MCU homologs in HeLa cells**. Immunoblot analysis of whole cell lysates from wild-type (WT) (**a**) and MCU knock-down (shMCU) (**b**) HeLa mt-AEQ cells stably expressing human or fungal MCU proteins fused to a C-terminal V5 tag using the following antibodies: α-MCU (Sigma Aldrich, HPA01648), α-V5 (Life Technologies, R96025), α-EMRE (Santa Cruz Biotechnology, sc- 86337), α-ACTIN (Sigma-Aldrich, A2228). NI, not infected. **c**, Analysis of protein topology by proteinase K (PK) treatment of mitochondria isolated from sh-MCU HeLa mt-AEQ cells expressing human and fungal MCU homologs, using the following antibodies: α-V5 (Life Technologies, R96025), α-TIM23 (BD Bioscience, 611222), α-TOM20 (Abcam, ab56783), and α-Cyclophilin D (Cyp D) (Abcam, ab110324). TOM20, TIM23, and Cyp D were used as controls for integral mitochondrial outer membrane, inner membrane and soluble matrix targeted proteins, respectively. T, triton (1%); Dig., digitonin. Source data are provided as a Source Data file.

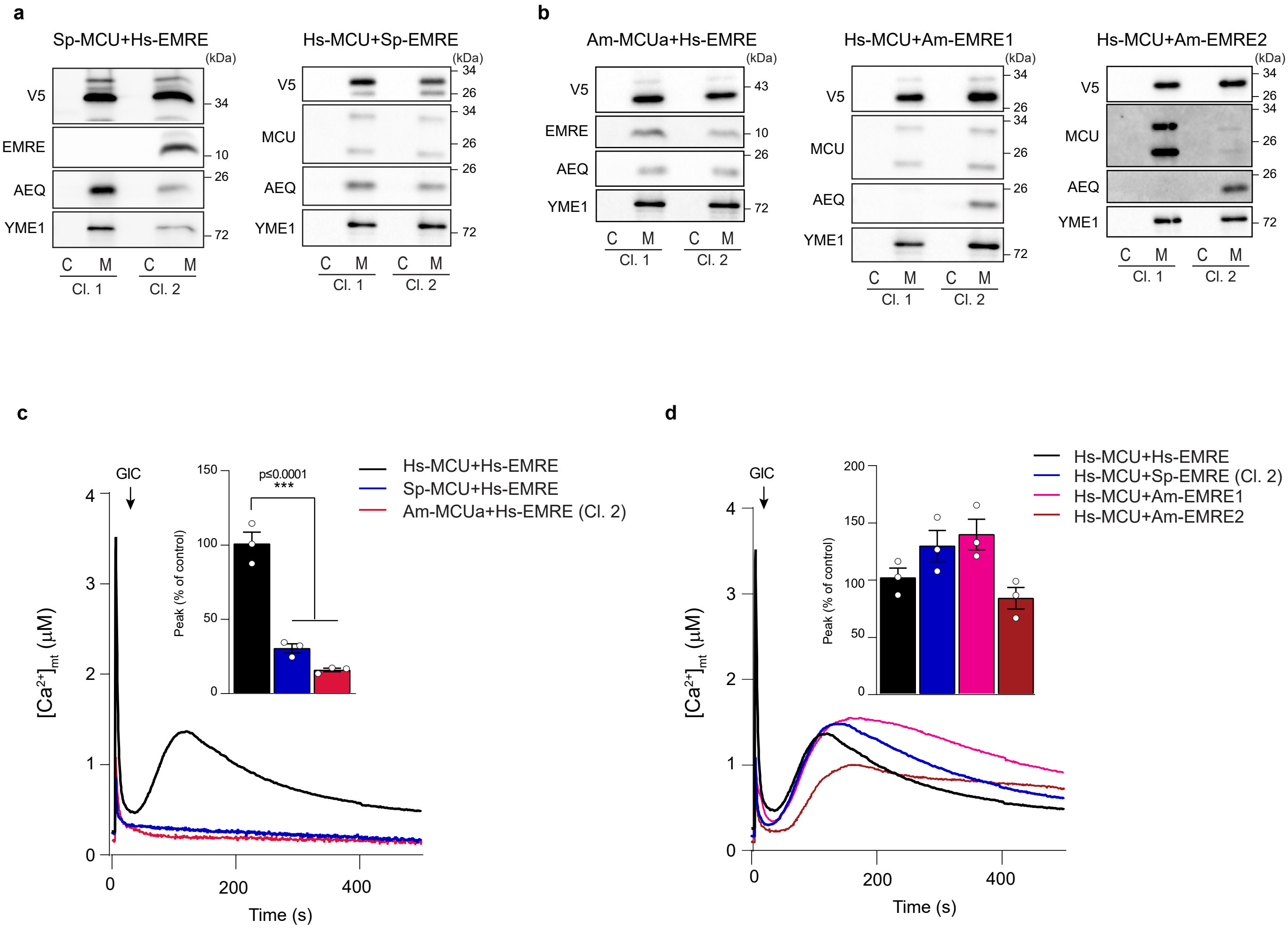

Supplementary Fig. 9. Reconstitution of mt-Ca^{2+} uptake in yeast cells expressing human MCU and EMRE with either fungal EMRE or MCU orthologs from *S. punctatus* and *A. macrogynus* respectively. **a**,**b**, Immunoblot analysis of cytosolic (C) and mitochondrial (M) fractions isolated from yeast clones (Cl.) expressing mt-AEQ together with human and either (**a**) *S. punctatus* or (**b**) *A. macrogynus* MCU and EMRE orthologs fused to a C-terminal V5-tag using the following antibodies: α-V5 (Life Technologies, R96025), α-MCU (Sigma Aldrich, HPA01648), α-EMRE (Santa Cruz Biotechnology, sc-86337), α-AEQ (Merck/Millipore, MAB4405), α-YME1. YME1 was used as control for yeast mitochondrial targeted protein. **c**,**d**, Representative traces and quantification of mt-Ca^{2+} transients in yeast cells expressing either MCU (**c**) or EMRE (**d**) orthologs from *S. punctatus* and *A. macrogynus* with human EMRE (Hs-EMRE) or MCU (Hs-MCU) respectively upon glucose-induced calcium (GIC) stimulation in presence of 1 mM $CaCl_2$ (n=3). All data represent mean ± SEM. *P* values are indicated in the different panels (**c**: ***p < 0.001, one-way ANOVA with Dunnett's Multiple Comparisons Test; **d**: *p = 0.036, one-way ANOVA with Dunnett's Multiple Comparisons Test). Source data are provided as Source Data file.

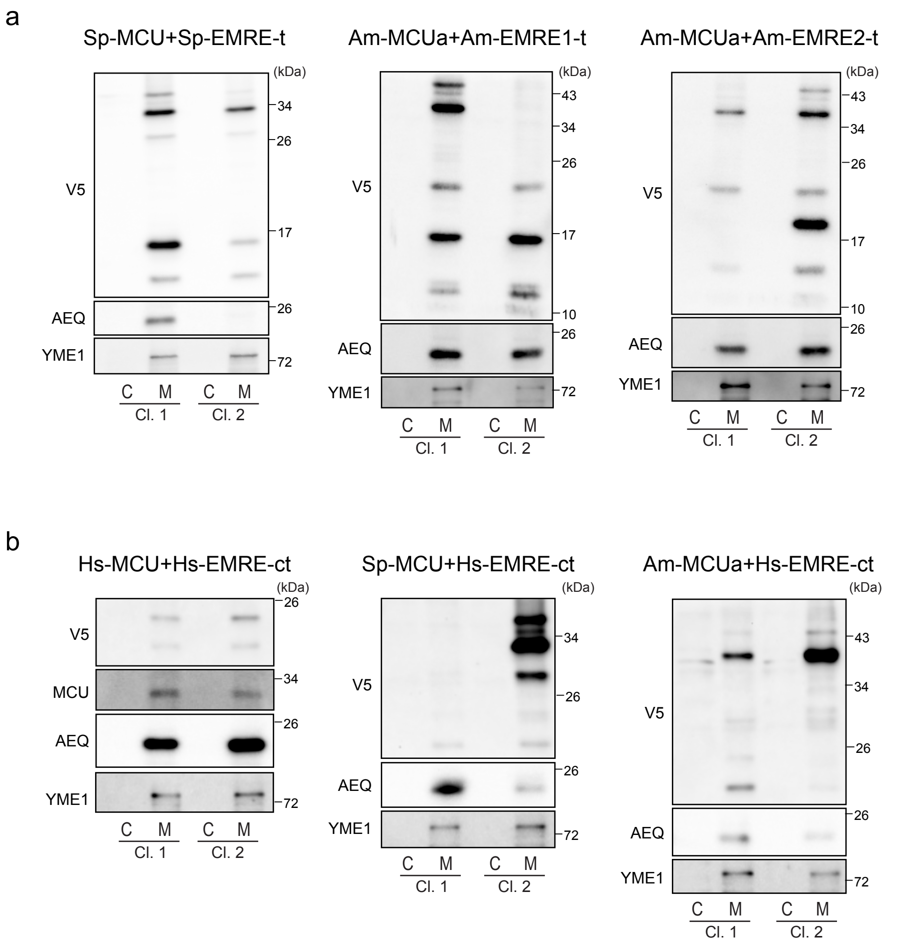

Supplementary Fig. 10. Heterologous expression of *S. punctatus* and *A. macrogynus* MCU and EMRE orthologs in yeast. a,**b**, Immunoblot analysis of cytosolic (C) and mitochondrial (M) fractions isolated from yeast clones (Cl.) expressing mt-AEQ together with either (**a**) *S. punctatus* (Sp-MCU) and *A. macrogynus* (Am-MCUa) MCU and their respective truncated EMRE (Sp-EMRE-t, Am-EMRE1-t, Am-EMRE2-t) or (**b**) Human, *S. punctatus* (Sp-MCU) *and A. macrogynus* (Am-MCUa) MCU and human EMRE with an added fungal extra C-terminal domain (Hs-EMRE-ct), fused to a C-terminal V5-tag, using the following antibodies: α-V5 (Life Technologies, R96025), α-AEQ (Merck/Millipore, MAB4405), α-YME1. YME1 was used as control for yeast mitochondrial targeted protein. Source data are provided as a Source Data file.

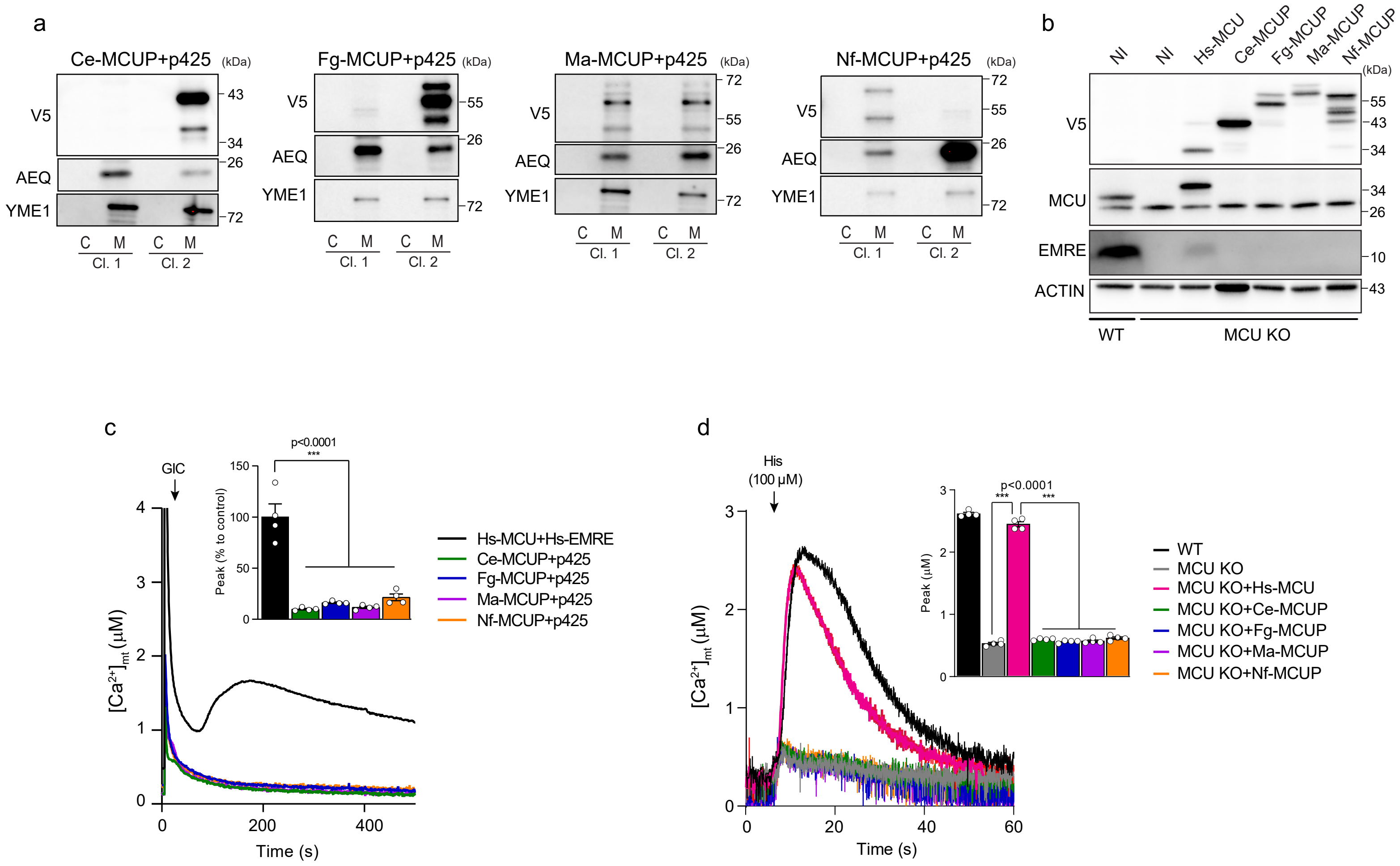

Supplementary Fig. 11. Reconstitution of mt-Ca^{2+} uptake in yeast and HeLa cells expressing fungal MCU paralogs. **a**, Immunoblot analysis of cytosolic (C) and mitochondrial (M) fractions isolated from yeast clones (Cl.) expressing mt-AEQ together with *C. europaea* (Ce-MCUP), *F. graminearum* (Fg-MCUP), *M. acridum* (Ma-MCUP) or *N. fischeri* (Nf-MCUP) MCU paralog fused to a C-terminal V5-tag and an empty vector (p425) using the following antibodies: α-V5 (Life Technologies, R96025), α-AEQ (Merck/Millipore, MAB4405), α-YME1. YME1 was used as control for yeast mitochondrial targeted protein. **b**, Immunoblot analysis of whole cell lysates from wild-type (WT) or MCU knock-out (MCU KO) HeLa mt-AEQ cells stably expressing human or fungal MCU homolog proteins fused to a C-terminal V5 tag using the following antibodies: α-MCU (Sigma Aldrich, HPA01648), α-V5 (Life Technologies, R96025), α-EMRE (Santa Cruz Biotechnology, sc- 86337), α-ACTIN (Sigma-Aldrich, A2228). NI, not infected. **c**, Representative traces and quantification of mt-Ca^{2+} transients in yeast cells expressing either human MCU (Hs-MCU) and EMRE (Hs-EMRE) or fungal MCU paralogs (Ce-MCUP, Fg-MCUP, Ma-MCUP or Nf-MCUP) and an empty vector (p425) upon glucose-induced calcium (GIC) stimulation in presence of 1 mM $CaCl_2$ (n=4). **d**, Quantification of mt-Ca^{2+} transients in WT or MCU KO HeLa mt-AEQ cells expressing different fungal MCU paralogs (Ce-MCUP, Fg-MCUP, Ma-MCUP or Nf-MCUP) upon histamine (His) stimulation (n=4). All data represent mean ± SEM. *P* values are indicated in the different panels (**c-d**: ***p < 0.0001, one-way ANOVA with Dunnett's Multiple Comparisons Test). Source data are provided as Source Data file.

Supplementary Figure 12 Uncropped Original Scans

Uncropped immunoblots from Supplementary Figure 4

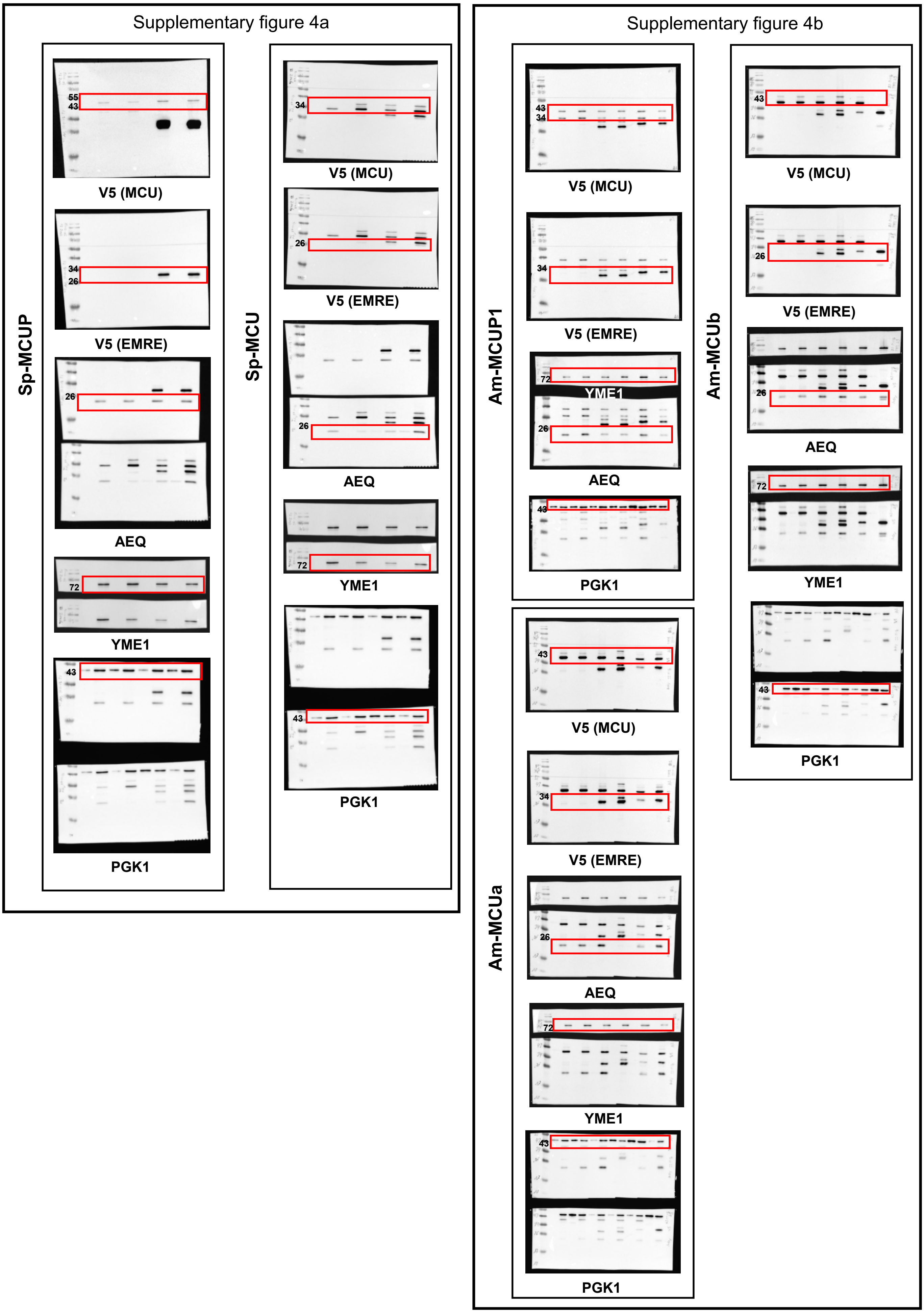

Uncropped immunoblots from Supplementary Figure 8

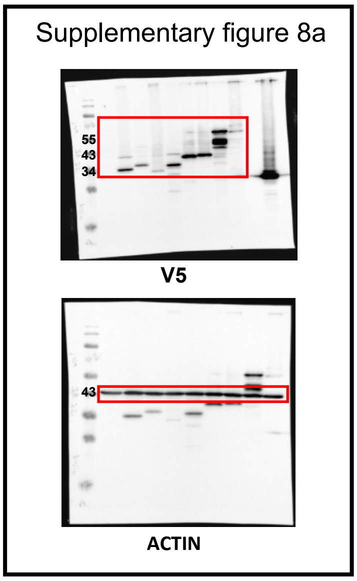

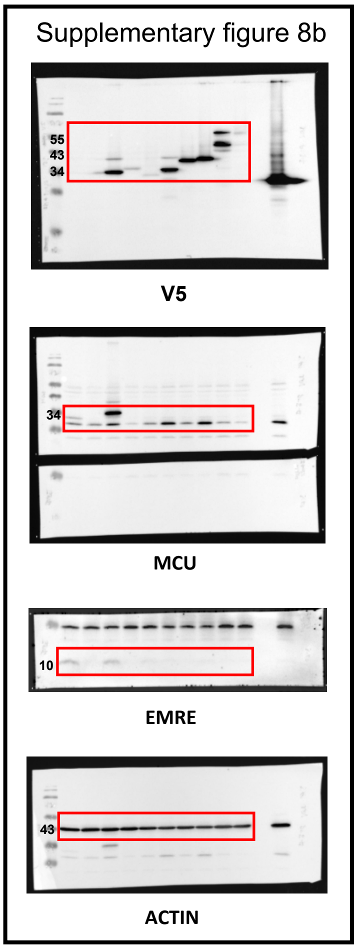

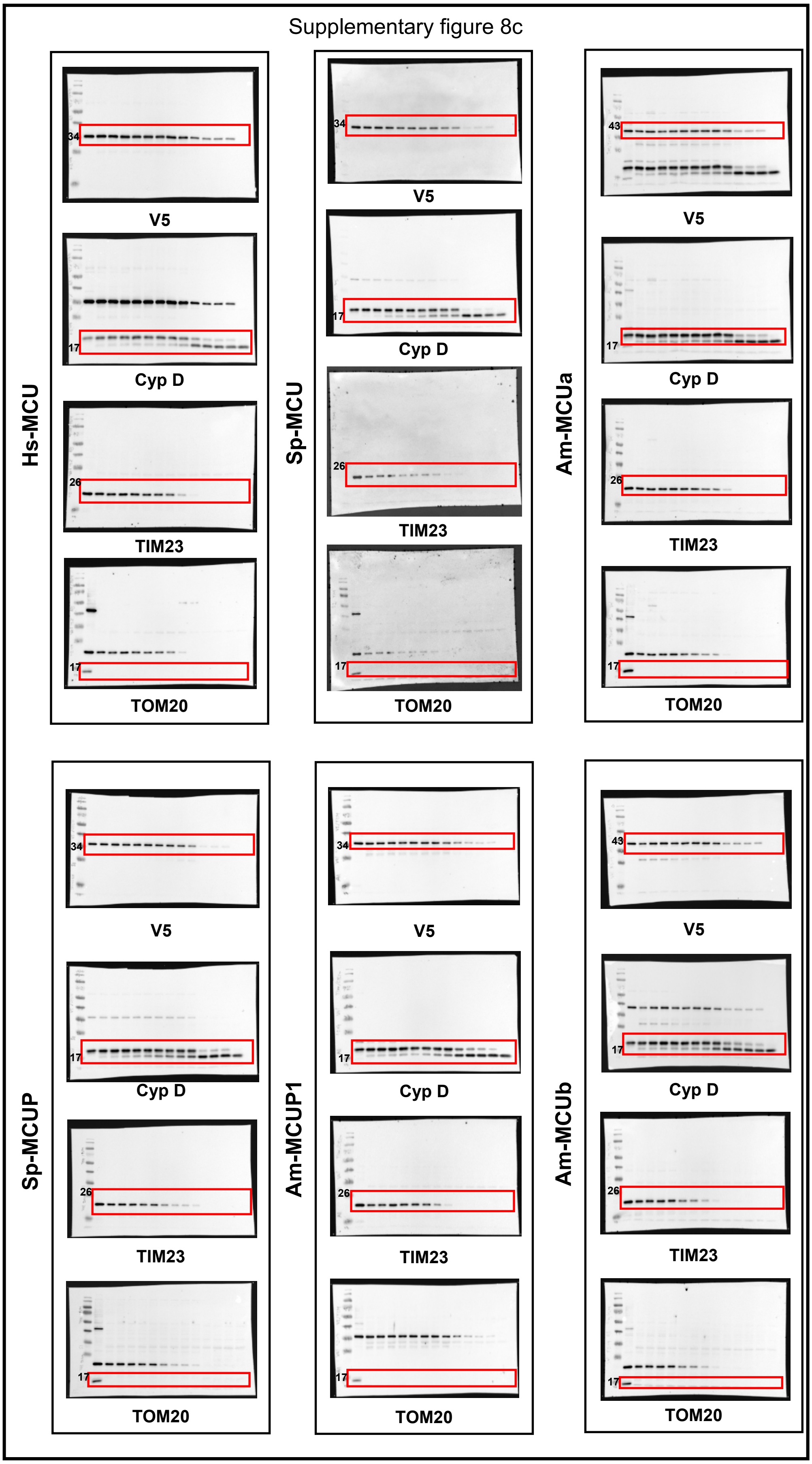

Uncropped immunoblots from Supplementary Figure 9

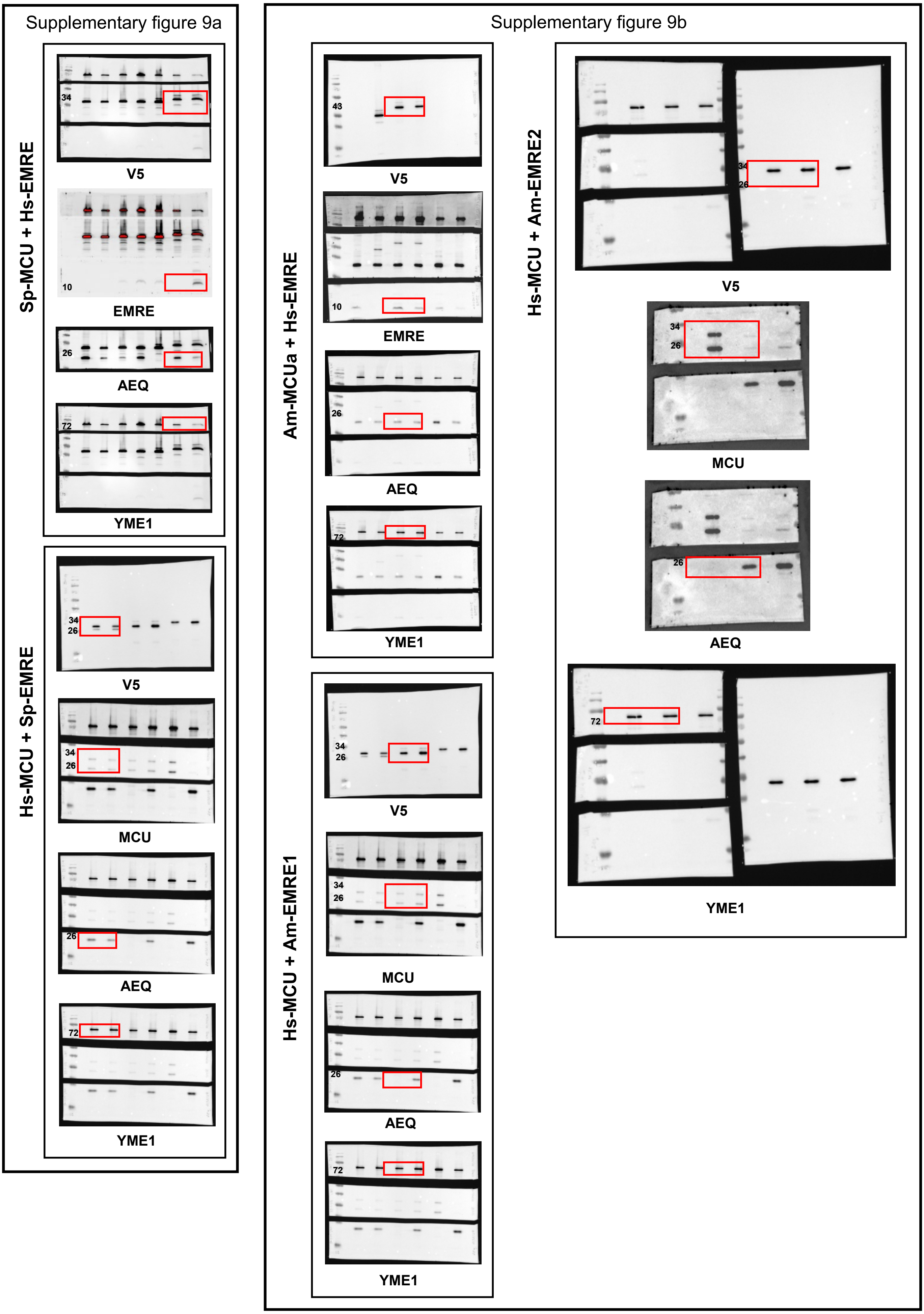

Uncropped immunoblots from Supplementary Figure 10

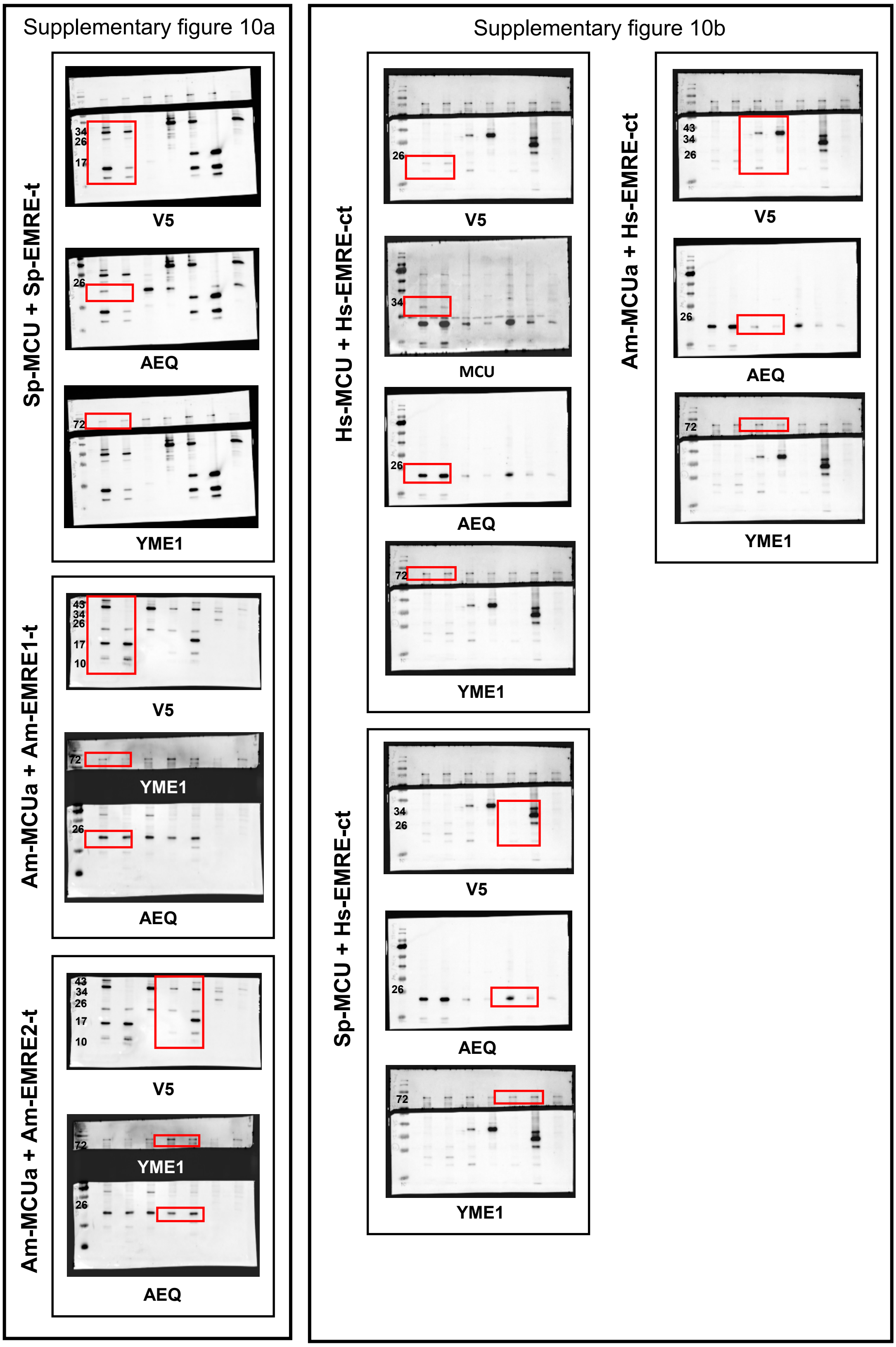

Uncropped immunoblots from Supplementary Figure 11

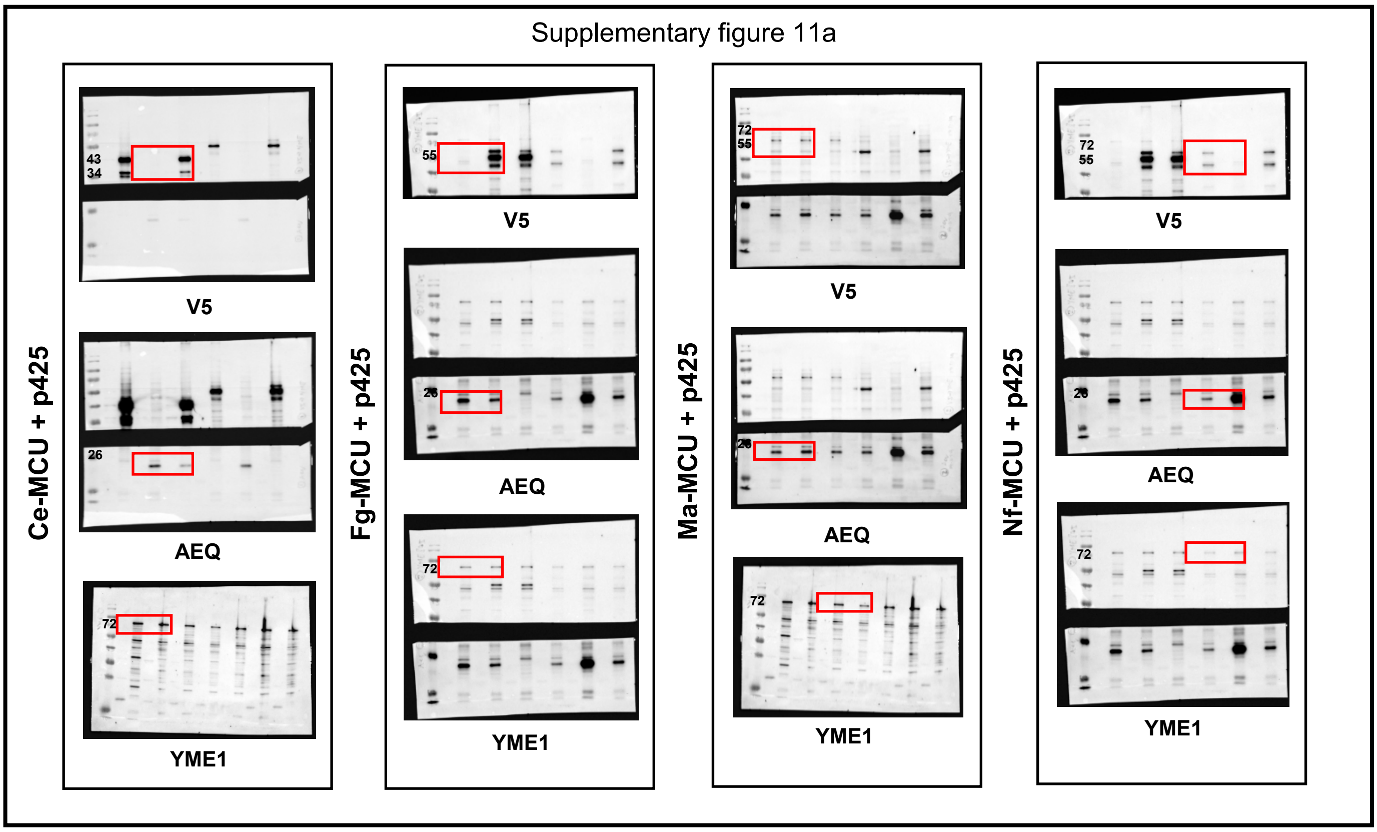

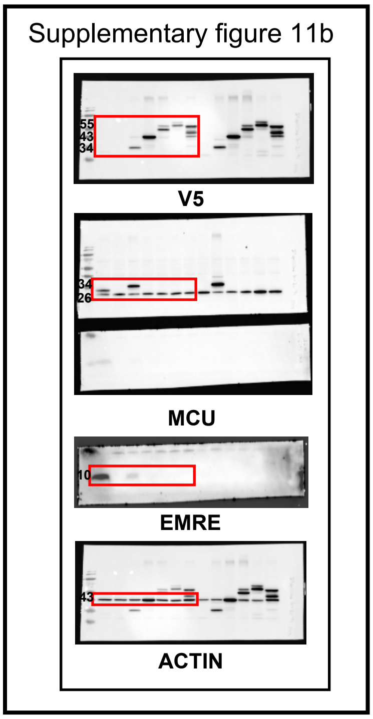

Supplementary Table 1. List of PCR primers used to amplify cDNA from PuC57 vector and perform Gateway cloning into pLX304 destination vector.

Species	cDNA	Direction	Primer sequence
Spizellomyces punctatus	Sp-MCU	Forward	5'-GGG GAC AAG TTT GTA CAA AAA AGC AGG CTT AGC CAC CAT GCG AAT CCC CTT CCA CCA CGG C-3'
		Reverse	5'-GGG GAC CAC TTT GTA CAA GAA AGC TGG GTT TGT TGC CTC CAC CCT GAC CC-3'
	Sp-MCUP	Forward	5'-GGG GAC AAG TTT GTA CAA AAA AGC AGG CTT AGC CAC CAT GGT GAG CCT GAT TCC AGT GGG GG-3'
		Reverse	5'-GGG GAC CAC TTT GTA CAA GAA AGC TGG GTT AAT CAG TCT GCC CAT TCG TCC AGC-3'
	Sp-EMRE	Forward	5'-GGG GAC AAG TTT GTA CAA AAA AGC AGG CTT AGC CAC CAT GTC CCG CAT TCT GAC ACG ATT CCC A-3'
		Reverse	5'-GGG GAC CAC TTT GTA CAA GAA AGC TGG GTT CCA GAA CTT CAG CCA GCC CCA-3'
	Sp-EMRE-t	Forward	5'-GGG GAC AAG TTT GTA CAA AAA AGC AGG CTT AGC CAC CAT GTC CCG CAT TCT GAC ACG ATT C-3'
		Reverse	5'-GGG GAC CAC TTT GTA CAA GAA AGC TGG GTT CAC CAC CTT CTC ATC GTC ATC CTT GT-3'
Allomyces macrogynus	Am-MCUa	Forward	5'-GGG GAC AAG TTT GTA CAA AAA AGC AGG CTT AGC CAC CAT GCT GTC AAG GGC TCT GCA GGT CG-3'
		Reverse	5'-GGG GAC CAC TTT GTA CAA GAA AGC TGG GTT CCC CTG CTT GCC CTC GCT TGT-3'
	Am-MCUb	Forward	5'-GGG GAC AAG TTT GTA CAA AAA AGC AGG CTT AGC CAC CAT GCT GAT TTC TTG TCG CCT GCT GGC T-3'
		Reverse	5'-GGG GAC CAC TTT GTA CAA GAA AGC TGG GTT TGA CTG CTG CTT TCC TGC GG-3'
	Am-MCUP1	Forward	5'-GGG GAC AAG TTT GTA CAA AAA AGC AGG CTT AGC CAC CAT GTT CGC ACA GTC CCG CCC ATT T-3'
		Reverse	5'-GGG GAC CAC TTT GTA CAA GAA AGC TGG GTT TTT GCC GCC CAG AAT CTC GTG-3'
	Am-EMRE1	Forward	5'-GGG GAC AAG TTT GTA CAA AAA AGC AGG CTT AGC CAC CAT GCC TCA GCT GCA TTT CTC ATC ATC TTT CG-3'
		Reverse	5'-GGG GAC CAC TTT GTA CAA GAA AGC TGG GTT CCA CCA TCG CAG GCT ATT TGA CCC-3'
	Am-EMRE2	Forward	5'-GGG GAC AAG TTT GTA CAA AAA AGC AGG CTT AGC CAC CAT GCC CCC TCT GCA CCA CGC C-3'
		Reverse	5'-GGG GAC CAC TTT GTA CAA GAA AGC TGG GTT CCA CCA CCT CCA ATG GCT TTT CC-3'
	Am-EMRE1-t	Forward	5'-GGG GAC AAG TTT GTA CAA AAA AGC AGG CTT AGC CAC CAT GCC TCA GCT GCA TTT CTC ATC ATC-3'
		Reverse	5'-GGG GAC CAC TTT GTA CAA GAA AGC TGG GTT CAC TCC TGC GTC ATC GTC ATC GT-3'
	Am-EMRE2-t	Forward	5'-GGG GAC AAG TTT GTA CAA AAA AGC AGG CTT AGC CAC CAT GCC CCC TCT GCA CCA CG-3'

		Reverse	5'-GGG GAC CAC TTT GTA CAA GAA AGC TGG GTT CAC TCC TGC GTC CTC ATC GTC A-3'
Homo sapiens	Hs-EMRE-ct	Forward	5'-GGG GAC AAG TTT GTA CAA AAA AGC AGG CTT AGC CAC CAT GGC GTC CGG AGC GGC-3'
		Reverse	5'-GGG GAC CAC TTT GTA CAA GAA AGC TGG GTT CCA GAA CTT CAG CCA GCC CC-3'
Cyphellophora europaea	Ce-MCUP	Forward	5'-GGG GAC AAG TTT GTA CAA AAA AGC AGG CTT AGC CAC CAT GAC TAA AGG CAA GCT GTT GAC GAC-3'
		Reverse	5'-GGG GAC CAC TTT GTA CAA GAA AGC TGG GTT TCT TGG TTC CGT TGT TGT TCT TTC GC-3'
Fusarium graminearum	Fg-MCUP	Forward	5'-GGG GAC AAG TTT GTA CAA AAA AGC AGG CTT AGC CAC CAT GAA CCA CGC TCT AAG GCG C-3'
		Reverse	5'-GGG GAC CAC TTT GTA CAA GAA AGC TGG GTT CGG CCA GGG CCG CAA-3'
Metarhizium acridum	Ma-MCUP	Forward	5'-GGG GAC AAG TTT GTA CAA AAA AGC AGG CTT AGC CAC CAT GGG CCA TGT CTT GGG TGG-3'
		Reverse	5'-GGG GAC CAC TTT GTA CAA GAA AGC TGG GTT GGT CCC AGC CCA TAT CGG TGT-3'
Neosartorya fischeri	Nf-MCUP	Forward	5'-GGG GAC AAG TTT GTA CAA AAA AGC AGG CTT AGC CAC CAT GCG GGC GCT TGT TAG CC-3'
		Reverse	5'-GGG GAC CAC TTT GTA CAA GAA AGC TGG GTT GCG CGT CAC ACT CAT GCT TGA-3'

Supplementary Table 2. List of PCR primers used to amplify cDNAs from pLX304 vector to perform cloning into yeast expression vectors.

Species	cDNA	Direction	Primer sequence
Spizellomyces punctatus	Sp-MCU	Forward	5'- GGG GGA TCC ATG CGA ATC CCC TTC CA-3'
	Sp-MCUP	Forward	5'- GGG GGA TCC ATG GTG AGC CTG ATT CCA G-3'
	Sp-EMRE	Forward	5'- GGG GGA TCC ATG TCC CGC ATT CTG ACA -3'
Allomyces macrogynus	Am-MCUa	Forward	5'- GGG GGA TCC ATG CTG TCA AGG GCT CTG-3'
	Am-MCUb	Forward	5'- GGG GGA TCC ATG CTG ATT TCT TGT CGC C-3'
	Am-MCUP1	Forward	5'- GGG GGA TCC ATG TTC GCA CAG TCC CG-3'
	Am-EMRE1	Forward	5'- GGG GGA TCC ATG CCT CAG CTG CAT TTC T-3'
	Am-EMRE2	Forward	5'- GGG GGA TCC ATG CCC CCT CTG CAC-3'
Cyphellophora europaea	Ce-MCUP	Forward	5'-GGG GGA TCC ATG ACC AAG GGC AAG CT-3'
Fusarium graminearum	Fg-MCUP	Forward	5'-GGG GGA TCC ATG AAC CAC GCC CTG AG-3'
Metarhizium acridum	Ma-MCUP	Forward	5'-GGG GGA TCC ATG GGA CAC GTG CTG G-3'
Neosartorya fischeri	Nf-MCUP	Forward	5'-GGG GGA TCC ATG AGA GCC CTG GTG TCT-3'
Homo sapiens	Hs-EMRE	Forward	5'-GGG CCC GGG ATG GCG TCC GGA GC-3'
	V5	Reverse	5'-GGG CTC GAG CTA CGT AGA ATC GAG ACC GAG-3'
