# Supplementary material for: Discovery of EMRE in fungi resolves the true evolutionary history of the mitochondrial calcium uniporter
Source: Nat Commun. 2020 Aug 12;11:4031. doi: 10.1038/s41467-020-17705-4 (PMC7423614; doi:10.1038/s41467-020-17705-4)
Supplement: Supplementary file 3 — Reporting Summary [file 41467_2020_17705_MOESM3_ESM.pdf]

Corresponding author(s): Fabiana Perocchi & Toni Gabaldón

Last updated by author(s): Jun 25, 2020

Reporting Summary

Nature Research wishes to improve the reproducibility of the work that we publish. This form provides structure for consistency and transparency in reporting. For further information on Nature Research policies, see our Editorial Policies and the Editorial Policy Checklist.

Statistics

For all statistical analyses, confirm that the following items are present in the figure legend, table legend, main text, or Methods section.

n/a	Confirmed	
☐	☒	The exact sample size (*n*) for each experimental group/condition, given as a discrete number and unit of measurement
☐	☒	A statement on whether measurements were taken from distinct samples or whether the same sample was measured repeatedly
☐	☒	The statistical test(s) used AND whether they are one- or two-sided<br>*Only common tests should be described solely by name; describe more complex techniques in the Methods section.*
☐	☒	A description of all covariates tested
☐	☒	A description of any assumptions or corrections, such as tests of normality and adjustment for multiple comparisons
☐	☒	A full description of the statistical parameters including central tendency (e.g. means) or other basic estimates (e.g. regression coefficient) AND variation (e.g. standard deviation) or associated estimates of uncertainty (e.g. confidence intervals)
☐	☒	For null hypothesis testing, the test statistic (e.g. *F*, *t*, *r*) with confidence intervals, effect sizes, degrees of freedom and *P* value noted<br>*Give P values as exact values whenever suitable.*
☒	☐	For Bayesian analysis, information on the choice of priors and Markov chain Monte Carlo settings
☒	☐	For hierarchical and complex designs, identification of the appropriate level for tests and full reporting of outcomes
☒	☐	Estimates of effect sizes (e.g. Cohen's *d*, Pearson's *r*), indicating how they were calculated

Our web collection on statistics for biologists contains articles on many of the points above.

Software and code

Policy information about availability of computer code

Data collection	No software was used for data collection.
Data analysis	Software that was used in the computational analyses of the study:<br>- HMMER 3.1b2<br>- NCBI Blast 2.7.1<br>- MAFFT v7.394<br>- IQ-TREE v1.6.8<br>- ETE3 (ETE toolkit)<br>- Jalview2<br>- Inkscape 0.92<br>- GraphPad Prism 8.0<br>- MATLAB R2014b<br><br>There is no specific new code developed for this project, custom python scripts were used to run analysis or parse results of the different programs, as described in the methods. These are available with no restriction under request.

For manuscripts utilizing custom algorithms or software that are central to the research but not yet described in published literature, software must be made available to editors and reviewers. We strongly encourage code deposition in a community repository (e.g. GitHub). See the Nature Research guidelines for submitting code & software for further information.

Data

Policy information about availability of data

All manuscripts must include a data availability statement. This statement should provide the following information, where applicable:

- Accession codes, unique identifiers, or web links for publicly available datasets
- A list of figures that have associated raw data
- A description of any restrictions on data availability

All genome data and predicted peptide sets are publicly available and were downloaded from Ensembl-v91 (https://www.ensembl.org/), and Ensembl fungi (https://fungi.ensembl.org/), metazoa(https://metazoa.ensembl.org/), plants (https://plants.ensembl.org/) and protists (https://protists.ensembl.org/index.html) v37.
The raw HMM profiles of the different protein families were downloaded from Pfam release 30.0 (https://pfam.xfam.org/).
Hs-MICU1 (MICU1_HUMAN) and NCLX (NCLX_HUMAN) sequences were downloaded from the Uniprot database (https://www.uniprot.org/uniprot/Q9BPX6 and https://www.uniprot.org/uniprot/Q6J4K2)
Source data for Figures 3c-d, 4c-f, Supplementary figures 3-5 and 7-11 have been provided in the Source Data File.
The data that support the findings of this study have no restriction and are available from the corresponding authors upon reasonable request.

Field-specific reporting

Please select the one below that is the best fit for your research. If you are not sure, read the appropriate sections before making your selection.

☒ Life sciences ☐ Behavioural & social sciences ☐ Ecological, evolutionary & environmental sciences

For a reference copy of the document with all sections, see nature.com/documents/nr-reporting-summary-flat.pdf

Life sciences study design

All studies must disclose on these points even when the disclosure is negative.

Sample size	Sample size was chosen based on previous publications to generate statistically significant results (reference: Wettmarshausen et al., 2018 and Arduino et al., 2017).
Data exclusions	No data was excluded from analsyes.
Replication	All attempts at replication were successful, obtaining the same experimental outcome at each replication.
Randomization	Samples were randomized in the measurement plates to ensure different samples are measured at the same time point.
Blinding	Blinding was not relevant to the study as all cells/samples were collected and analyzed in the same unbiased way.

Reporting for specific materials, systems and methods

We require information from authors about some types of materials, experimental systems and methods used in many studies. Here, indicate whether each material, system or method listed is relevant to your study. If you are not sure if a list item applies to your research, read the appropriate section before selecting a response.

Materials & experimental systems

n/a	Involved in the study	
☐	☒	Antibodies
☐	☒	Eukaryotic cell lines
☒	☐	Palaeontology and archaeology
☒	☐	Animals and other organisms
☒	☐	Human research participants
☒	☐	Clinical data
☒	☐	Dual use research of concern

Methods

n/a	Involved in the study	
☒	☐	ChIP-seq
☒	☐	Flow cytometry
☒	☐	MRI-based neuroimaging

Antibodies

Antibodies used	Mouse monoclonal anti-ß-Actin (Sigma-Aldrich; Cat#A2228; Lot#085M4754V; RRID: AB_476697)<br>Mouse monoclonal anti-TOMM20 (Abcam; Cat#Ab56783; Lot#GR3188177-1; RRID: AB_945896)<br>Mouse monoclonal anti-V5 (Life Technologies; Cat#R96025; Lot#1792242; RRID: AB_2556564)<br>Mouse monoclonal anti-Cyclophilin D [E11AE12BD4] (Abcam; Cat#ab110324; Lot#GR134866-15; RRID: AB_10864110)<br>Mouse monoclonal anti-TIM23 (BD Bioscience; Cat#611222; Lot#3067849; RRID: AB_398754)<br>Anti-Sc-Yme1 produced in rabbit (Schreiner et al., 2012)

	Mouse monoclonal anti-Aequorin (clone 6E3.2) (Merck; Cat#MAB4405; RRID: AB_94900; RRID: AB_94900)<br>Rabbit polyclonal anti-EMRE - C22orf32 (clone C-12) (Santa Cruz Biotechnology; Cat#sc-86337; Lot#K0215; RRID: AB_2250685)<br>Rabbit polyclonal anti-MCU (Sigma-Aldrich; Cat#HPA016480; Lot#C0114358; RRID: AB_2071893)<br>Mouse monoclonal anti-PGK1 (Life Technologies; Cat# 459250; Lot#UD2749418; RRID: AB_2532235)
Validation	Antibody validation was deferred to the manufacturers and was supported by multiple publication.<br>All the primary antibodies used in the study have been previously validated on yeast and mammalian cells and mitochondria (reference: Wettmarshausen et al., 2018 and Arduino et al., 2017). Each primary antibody was validated by immunoblotting cell lysates from knock-down, knock-out or heterologous expression of the respective proteins that are targeted by the antibody in yeast and mammalian HeLa cells, obtaining protein bands that are of similar molecular weight to their respective protein sizes.

Eukaryotic cell lines

Policy information about cell lines

Cell line source(s)	All mammalian cell lines and yeast strains have been generated for this paper, except for the following:<br>pLKO HeLa cells stably expressing WT mt-AEQ; shMCU HeLa cells stably expressing WT mt-AEQ; shMCU HeLa cells stably expressing WT mt-AEQ + HsMCU; shMCU HeLa cells stably expressing WT mt-AEQ + AfMCU; shMCU HeLa cells stably expressing WT mt-AEQ + NcMCU (source: Wettmarshausen et al., 2018).<br>S. cerevisiae: Strain background: YPH499 expressing HsMCU + HsEMRE + WT mt-AEQ (Source: Arduino et al., 2017).
Authentication	All mammalian cell lines and yeast strains used in this study were authenticated by PCR and western blot analysis
Mycoplasma contamination	All mammalian cell lines have been tested negative for mycoplasma contamination.
Commonly misidentified lines<br>(See ICLAC register)	No commonly misidentified cell lines were used in the study.
