# Supplementary material for: Discovery of EMRE in fungi resolves the true evolutionary history of the mitochondrial calcium uniporter
Source: Nat Commun. 2020 Aug 12;11:4031. doi: 10.1038/s41467-020-17705-4 (PMC7423614; doi:10.1038/s41467-020-17705-4)
Supplement: Supplementary file 4 — Description of Additional Supplementary Files [file 41467_2020_17705_MOESM4_ESM.pdf]

Description of Additional Supplementary files

File name: Supplementary Data 1

Description: List of genomic datasets used, genome assembly codes, and protein ids of MCU, MICU, EMRE, MCUP, and NCLX sequences in each species.

File name: Supplementary Data 2

Description: Raw phylogenetic trees in newick format of MCU and MICU families (Figure 2), and NCLX (not shown).
