# Supplementary material for: Discovery of EMRE in fungi resolves the true evolutionary history of the mitochondrial calcium uniporter
Source: Nat Commun. 2020 Aug 12;11:4031. doi: 10.1038/s41467-020-17705-4 (PMC7423614; doi:10.1038/s41467-020-17705-4)
Supplement: Supplementary file 2 — Peer Review [file 41467_2020_17705_MOESM2_ESM.pdf]

Editorial Note: This manuscript has been previously reviewed at another journal that is not operating a transparent peer review scheme. This document only contains reviewer comments and rebuttal letters for versions considered at *Nature Communications.*

Reviewer #3 (Remarks to the Author):

The authors provided additional experimental data to address my original concerns. I think the manuscript is improved and experimental data fully support the conclusion. I think the paper is now ready for publication.

Reviewer #4 (Remarks to the Author):

In their paper, Pittis, Goh et al. present a comprehensive phylogenetic analysis of the mitochondrial calcium uptake pathway - the MCU, MICU1 and EMRE gene families - across eukaryotes. They use this analysis to identify bona-fide EMRE and MICU1 orthologs in chytrid fungi; trace the lack of calcium uptake activity in fungal-specific MCU paralogs to an ancient duplication in early opisthokonts; and use ectopic expression of various MCU and EMRE orthologues to experimentally verify their phylogenetic predictions.

This is a thorough, well-executed study that merits publication.
The revised paper is much better and the new fIgures are, for the most part, excellent.

Certain sections of the text and analysis, however, would benefit from a rewrite to better convey the authors' findings to the reader and to avoid confusion.

Specifically:

*As before, the emphasis on the "evolutionary paradox" is distracting. Is isn't much of a paradox (or surprise) that a deeply conserved gene family, with a complicated history of losses and duplications, has functionally diverged in one particular section of the tree.

*The authors would be better served starting the paper up-front with a description of MCU, MICU1 and EMRE function, leading on to the phylogenetic distributions and the trees. Currently readers are left not knowing what these proteins do. The same section should also explain how certain non-opisthokont MCUs, like the Dictyostelium or Nematostella variants, are able to mediate calcium uptake without MICU1. This leads to the question, what is the fungal MCU doing? And why is it different? This should be addressed.

*The section on the MCU and MICU1 trees (Figure 2) is confusing because it is not possible to trace the co-evolution of the families through the phylogenetic trajectories displayed separately in 2a and 2b. Since the rest of the paper only deals with the MCU-EMRE interaction (or lack thereof), what conclusion are we supposed to draw from the phylogenetic distribution of MICU1? What do the authors want readers to understand about MICU? Has MCIU diverged along the same path as MCU? Does the chytrid MICU1 function like the opisthokont MICU1? If amoebozoa have MICU1, how is the Dictyostelium MCU able to reconstitute calcium import by itself?

*Important: rather than using terms such as the "fungal animal-like" MCU etc, which is extremely confusing, the authors should define classes with names unrelated to their phylogenetic distributions at the beginning of the paper and retain those definitions throughout.

*The new last paragraph of text, explaining the functional relevance of detecting an older origin for EMRE and clarifying the nature EMRE-MCU interaction by making use of the chytrid version is very good and should be linked back to the introduction. Along the same lines, the new Figure 4 is excellent.

Minor:
*Figure 1: the red arrows pointing to the chytrid genomes could be labeled directly on the figure, it would help the reader

Reviewer #5 (Remarks to the Author):

Summary:
In this manuscript, Pittis et al. investigate the phylogenetic distributions of the core MCU complex components, including MCU, MICU, and EMRE across eukaryotes. The authors report two functionally different MCU paralogs in chytrid fungi, an animal-like MCU and fungal-specific MCU (fsMCU). This study identifies EMRE in three chytrid fungi (Allomyces macrogynus, Catenaria anguillulae, and Spizellomyces punctatus) that was previously identified only in metazoans and reported to be essential for MCU channel function. Employing a yeast reconstitution system, the authors show that animal-like MCU requires EMRE to facilitate mitochondrial Ca2+ uptake. In contrast, fsMCUs do not mediate mito-Ca2+ uptake, even in the presence of EMRE. The study demonstrates a tight co-evolution between MCU and EMRE proteins and offers a framework to understand the sequence determinants of this interaction.

Major Concerns:
1. The study is well designed from an evolutionary perspective, and phylogenetic analyses are clear. However, several groups have already reported the phylogenetic distribution, function, and structure of the MCU channel (Bick et al. Science. 2012; Nguyen et al. Nature. 2018; Fan et al. Nature 2018). It is also well known that EMRE is an essential regulator of the MCU channel (Sancak et al. Science. 2013.; Wang et al. Cell 2019; Liu et al. JCI Insight 2020). Given the limited novelty the authors failed to show how these studies will help to further our understanding of mitochondrial physiology. How do these results translate and inform our understanding of human mtCU function?

2. The study design lacks rigor. All the calcium measurements were performed using the photoprotein aequorin, which has many limitations and is not accepted as a gold-standard experimental approach. Numerous other fluorescent reporters are far superior and more is needed to define the mito-calcium exchange dynamics. This methodology issue is clear in Extended Data Fig. 7, where all the fungal MCUs and MCUBs can increase mito Ca2+ (~2.5uM peak) in response to histamine stimulation.

3. Some of the species (Sarcoptes scabiei, Trichoplax adhaerens, Perkinsus marinus ATCC 50983) do not have any MCU homolog, however, MICU1 is present (extended data). The MICU homolog in these species also has EF-hand domains. What is the relevance of MICU in these species, where MCU is not present?

4. You show that animal-like MCU co-expressed with Hs-EMRE in yeast is unable to reconstitute mito Ca2+ uptake, suggesting that the extended C-terminal of fungal EMRE is necessary to activate animal-like MCU. However, the chimeric hsEMRE with the C-terminal of fungal EMRE (hsEMREct), is able to activate Hs-MCU, but is unable to reconstitute the mito-Ca2+ uptake when co-expressed with animal-like MCU. This appears to be contradicting the hypothesis. The authors should perform additional experiments to validate the involvement of the fungal C-terminal domain in MCU activation. There is no biochemical evidence presented forthe animal-like MCU, fungal EMRE and fungal MICU1 interactions beyond the aequorin reporter.

5. In fungal EMRE, the MCU interacting domain GXXXA/S/G and the poly aspartate tail necessary for binding to MICU1 are fully conserved. Does hsEMREct still interact with fungal MCU and MICU1? These concerns can be easily addressed by co-immunoprecipitation experiments.

Minor:
1. Abbreviations should be mentioned in figure legends for all groups like “NI” and “pLKO” in Extended Data Fig. 7 is defined anywhere.

Reviewer #6 (Remarks to the Author):

General Comments: This is an important and novel study showing that previously identified animal and fungal MCUs, the targets of several structural and functional efforts, represent two distinct paralogous subfamilies originating from an ancestral duplication. The authors further show a complete “animal like” uniporter complex within chytrid fungi, including bona-fide orthologs of MCU, MICU1, and EMRE. This is a very important study for understanding the regulation of MCU. It is generally acknowledge that EMRE is only present in metazoans. Furthermore this study calls into question whether recent structural studies of fungal MCU (which this study suggested do not transport calcium) are an appropriate model to understand MCU mediated calcium transport in metazoans. This is a well done study and the authors have clearly addressed all the concerns raised in the previous review.
Specific Comments:
1. Fig 3b and 3c are very interesting. It might be nice to more explicitly in the text discuss that there are 2 EMRE genes in A. macrogynus (and indeed C. anguillulae in Fig 4b, though no data is shown for that species), and interestingly yeast reconstituted with the presence of MCU and each EMRE exhibit different degrees of calcium uptake (Fig. 3c).
2. Fig 5 is great for clarifying the main conclusion; however, lines 103-108 make it sound as though the common ancestor of opisthokonts had both animal-like and fungal-specific MCUs and then Holozoa and fungi each lost one and kept the other. Fig. 5 makes it look like the animal-like MCU came first and then later the fungi had a duplication event. Can these be reconciled?

Minor:
Based on how it's spelled everywhere else, "chytrid" is spelled wrong on line 116.

Line 144 and 146 - it might be clearer to refer to "chytrid" EMRE instead of "fungal" EMRE just to be more specific since the story suggests that most fungi lost "animal-like" MCU and EMRE, but up to the authors

Line 159 - saying "the presence of the extra C-terminal domain in Hs-EMRE" is confusing, please clarify that the extra chytrid C-terminal domain was fused to Hs-EMRE

Discovery of EMRE in fungi resolves the true evolutionary history of the mitochondrial calcium uniporter

Point-by-point response to the reviewers' comments

Response to reviewer #3:

The authors provided additional experimental data to address my original concerns. I think the manuscript is improved and experimental data fully support the conclusion. I think the paper is now ready for publication.

Response: We thank reviewer 3, and we are happy that he/she is satisfied with all the changes implemented over the previous version.

Response to reviewer #4:

In their paper, Pittis, Goh et al. present a comprehensive phylogenetic analysis of the mitochondrial calcium uptake pathway - the MCU, MICU1 and EMRE gene families - across eukaryotes. They use this analysis to identify bona-fide EMRE and MICU1 orthologs in chytrid fungi; trace the lack of calcium uptake activity in fungal-specific MCU paralogs to an ancient duplication in early opisthokonts; and use ectopic expression of various MCU and EMRE orthologues to experimentally verify their phylogenetic predictions.

This is a thorough, well-executed study that merits publication.
The revised paper is much better and the new figures are, for the most part, excellent.

Certain sections of the text and analysis, however, would benefit from a rewrite to better convey the authors' findings to the reader and to avoid confusion.

Response: We thank reviewer 4 for his/her comments. We have re-written some sections to better convey our findings, as suggested.

Specifically:

*As before, the emphasis on the "evolutionary paradox" is distracting. Is isn't much of a paradox (or surprise) that a deeply conserved gene family, with a complicated history of losses and duplications, has functionally diverged in one particular section of the tree.

Response: We agree with the reviewer that a complicated story of gains, losses, and functional divergence is not a surprise. With the "evolutionary paradox" we refer to the paradox that represented - before our study- the presence of MCU homologs in fungal organisms lacking MICU and mt-Ca^{2+} uptake activity. This finding was incongruent with knowledge at that time,

and it has been called a paradox in the literature (see Kamer KJ & Mootha VK, 2015. The molecular era of the mitochondrial calcium uniporter. Nature Reviews). Our results reconcile this observation with the true evolutionary history of the MCU family, by showing that only one branch of the family is expected to retain the function, and to co-evolve with MICU (across eukaryotes) and also EMRE (across opisthokonts). We now made an additional effort to make this clearer to the readers.

*The authors would be better served starting the paper up-front with a description of MCU, MICU1 and EMRE function, leading on to the phylogenetic distributions and the trees. Currently readers are left not knowing what these proteins do. The same section should also explain how certain non-opisthokont MCUs, like the Dictyostelium or Nematostella variants, are able to mediate calcium uptake without MICU1. This leads to the question, what is the fungal MCU doing? And why is it different? This should be addressed.

Response: We agree with the reviewer, and have now extended the introduction to better introduce the functions of these proteins. Regarding the lack of MICU1 in *Nematostella* and *Dyctiostelium*, we want to note that both have MICU, as shown in Extended Data Fig. 1. The reviewer perhaps refers to *Thelohanellus kitauei* -where MICU is indeed not detected, probably due to incompleteness of the genome or some other methodological issue- or to EMRE rather than MICU1 –which is not detected in the two species. While *Dictyostelium* is not expected to have an EMRE gene as a non-opisthokont, and MCU alone is sufficient to reconstitute mt-Ca^{2+} uptake, the MCU of the sea anemone *Nematostella* (Extended Data Fig. 5) was functional only when co-expressed with Hs-EMRE. Thus, the failure to detect EMRE in *Nematostella* should similarly be attributed to technical reasons. As we explain in the paper, MCU and EMRE together or MCU alone, in opisthokonts and non-opisthokonts respectively, are sufficient to form a Ca^{2+} conducting channel (Kovács-Bogdán et al., PNAS 2014; Tsai et al., Elife 2016; Wang et al., Cell 2019). MICU is not required for MCU-dependent Ca^{2+} transport, but it is necessary for regulating its gating and activation properties (Csordás et al., Cell Met 2013), and in fungi is only found in three chytrids with EMRE and functional “animal-like” MCU. We stress this better in the manuscript.

*The section on the MCU and MICU1 trees (Figure 2) is confusing because it is not possible to trace the co-evolution of the families through the phylogenetic trajectories displayed separately in 2a and 2b. Since the rest of the paper only deals with the MCU-EMRE interaction (or lack thereof), what conclusion are we supposed to draw from the phylogenetic distribution of MICU1? What do the authors want readers to understand about MICU? Has MICU diverged along the same path as MCU? Does the chytrid MICU1 function like the opisthokont MICU1? If amoebozoa have MICU1, how is the Dictyostelium MCU able to reconstitute calcium import by itself?

Response: As we mentioned, MICU is present in *Dictyostelium* (both *purpureum* and *discoideum*) as shown in Extended Data Fig. 1. In fact, our expectation is that MICU is present

in all MCU-encoding species, throughout eukaryotes. Across the 1156 genomes in our dataset the presence-absence overlap is almost perfect; in 1144 genomes MCU and MICU co-occur (both present or absent), and only in 12 cases one is undetected in the presence of the other (~1%), which is most probably due to methodological issues or incompleteness of some genomes (false negative). Given this striking co-evolution pattern across all lineages, we believe that it is reasonable to assume that while MICU is not necessary for a functional uniporter channel, it is essential for its physiological regulation. In Figure 2, we aimed to show that the co-evolution pattern is detected only if the "true" orthologous sequences are considered (excluding ARALAR homologs for MICU1 and the fungal paralogous clade for MCU). We now clarify this in the manuscript, Figure 2, and the legend accordingly.

*Important: rather than using terms such as the "fungal animal-like" MCU etc, which is extremely confusing, the authors should define classes with names unrelated to their phylogenetic distributions at the beginning of the paper and retain those definitions throughout.

Response: We now define the animal-like family as MCU, and the paralog as MCUP and use these names throughout.

*The new last paragraph of text, explaining the functional relevance of detecting an older origin for EMRE and clarifying the nature EMRE-MCU interaction by making use of the chytrid version is very good and should be linked back to the introduction. Along the same lines, the new Figure 4 is excellent.

Response: We thank the reviewer for his/her nice comments, we made an effort to link back that result with the introduction.

Minor:
*Figure 1: the red arrows pointing to the chytrid genomes could be labeled directly on the figure, it would help the reader

Response: Good idea, we did as suggested.

Response to reviewer #5:

Summary:
In this manuscript, Pittis et al. investigate the phylogenetic distributions of the core MCU complex components, including MCU, MICU, and EMRE across eukaryotes. The authors report two functionally different MCU paralogs in chytrid fungi, an animal-like MCU and fungal-specific MCU (fsMCU). This study identifies EMRE in three chytrid fungi (Allomyces macrogynus, Catenaria anguillulae, and Spizellomyces punctatus) that was previously identified only in metazoans and reported to be essential for MCU channel function. Employing a yeast reconstitution system, the authors show that animal-like MCU requires EMRE to facilitate

mitochondrial Ca2+ uptake. In contrast, fsMCUs do not mediate mito-Ca2+ uptake, even in the presence of EMRE. The study demonstrates a tight co-evolution between MCU and EMRE proteins and offers a framework to understand the sequence determinants of this interaction.

Response: We thank the reviewer for his/her comments.

Major Concerns:
1. The study is well designed from an evolutionary perspective, and phylogenetic analyses are clear. However, several groups have already reported the phylogenetic distribution, function, and structure of the MCU channel (Bick et al. Science. 2012; Nguyen et al. Nature. 2018; Fan et al. Nature 2018). It is also well known that EMRE is an essential regulator of the MCU channel (Sancak et al. Science. 2013.; Wang et al. Cell 2019; Liu et al. JCI Insight 2020). Given the limited novelty the authors failed to show how these studies will help to further our understanding of mitochondrial physiology. How do these results translate and inform our understanding of human mtCU function?

Response: Although it is indeed true that previous studies have assessed the phylogenetic distribution of MCU and EMRE's role in the channel, the implications of our results go well beyond just expanding the genomic datasets and testing proteins from a few extra species. Our evolutionary analysis -supported by direct experimental validation- manages to explain previously conflicting observations, and also reveals that the MCU-EMRE interaction exists beyond animals, providing the correct framework to understand it. A phylogenetic distribution on its own cannot resolve complex stories of duplication, and orthology and paralogy relationships, which is the most important addition of our study. Contrary to previous efforts, our comprehensive phylogenetic analysis serves to uncover a case of hidden paralogy, and points to distinct functionality of MCU homologs (actually paralogs as we show) in most fungi -that have wrongly been assumed to be equivalent and consequently used in many of the recent structural studies. The discovery of EMRE in basal fungi establishes the evolutionary origin of the interaction, and predicts that it should exist throughout the whole opisthokonta clade for organisms that have both EMRE and MCU. Therefore we strongly disagree with the notion that our work carries little novelty. We now stress these points even further in our revised manuscript.

2. The study design lacks rigor. All the calcium measurements were performed using the photoprotein aequorin, which has many limitations and is not accepted as a gold-standard experimental approach. Numerous other fluorescent reporters are far superior and more is needed to define the mito-calcium exchange dynamics.

Response: The reviewer's claim that the aequorin is "not accepted as a gold-standard experimental approach" is quite puzzling to us. Aequorin-based quantification of mitochondrial Ca^{2+} uptake was actually used to show for the first time that mitochondria do take up Ca^{2+} in vivo (Rizzuto et al., Nature 1992) and it has since been widely applied (Bonora et al., Nature Protocol 2013) – and therefore "accepted"- by many renown leading laboratories in the field of

Ca^{2+} signaling. This means that the use of aequorin for quantifying mitochondrial Ca^{2+} dynamics is instead fully validated. Indeed, in the seminal papers on the discovery of MCU (Baughmann et al., Nature 2011; De Stefani et al., Nature 2011) most of the Ca^{2+} measurements were performed using the photoprotein aequorin. Of course the probe has limitations, as all Ca^{2+} sensors do. We respect that the reviewer would rather use a different one, but this is a matter of "taste" and not of rigor.

This methodology issue is clear in Extended Data Fig. 7, where all the fungal MCUs and MCUBs can increase mito Ca2+ (~2.5uM peak) in response to histamine stimulation.

Response: It is clear that the reviewer has misunderstood the experimental results in Extended Data Fig. 7. In Ext. Data Fig. 7a, we report mt-Ca^{2+} uptake in HeLa cells infected with lentiviral particles expressing either Hs-MCU or fungal MCU homologs. Therefore, those proteins are expressed in addition to the endogenous Hs-MCU and Hs-EMRE. If the reviewer looks carefully, he/she would notice that un-infected control (NI), which refers to wild-type HeLa cells, shows a peak of about 2.5 uM. Thus, the fact that "all the fungal MCUs and MCUBs can increase mt-Ca^{2+}" to the same level as the control in response to histamine, clearly indicates that they do not affect the activity of endogenous Hs-MCU and MCU channels. Thus, they are not functional. Otherwise, we would expect a gain-of-function phenotype as in HeLa cells over-expressing Hs-MCU.

Instead, Ext. Fig. 7b reports mt-Ca^{2+} uptake in HeLa cells where MCU is stably knocked-down (sh-MCU). Here, we either re-introduce Hs-MCU or fungal MCU homologs (MCU and MCUP). This is a classical example of functional rescue. As expected, the re-introduction of Hs-MCU leads to a complete rescue of mt-Ca^{2+} uptake, which is similar to the un-infected wild-type HeLa cells (pLKO) and has an almost similar peak value to that of NI in Ext. Fig. 7a, further suggesting that the results are reproducible. Instead, unlike Hs-MCU, all the fungal MCUs and MCUPs were unable to rescue mt-Ca^{2+} uptake and had peak values similar to that of cells with MCU knock-down, due to the absence of a fungal EMRE.

3. Some of the species (Sarcoptes scabiei, Trichoplax adhaerens, Perkinsus marinus ATCC 50983) do not have any MCU homolog, however, MICU1 is present (extended data). The MICU homolog in these species also has EF-hand domains. What is the relevance of MICU in these species, where MCU is not present?

Response: Among 1156 genomes assessed in this study, MCU and MICU are both found or missing together in 1144 species. They do not coincide in only 12 cases (~1%). The latter are most probably due to incompleteness in the annotation of some genomes or in the detection of orthologs by similarity search methods (false negative). In this context the existence of exceptions to co-evolution patterns is not only understandable, but even expected. However, these few exceptions do not detract from the striking MCU-MICU co-evolution pattern across all lineages. We now clarify this in the text.

4. You show that animal-like MCU co-expressed with Hs-EMRE in yeast is unable to reconstitute mito Ca2+ uptake, suggesting that the extended C-terminal of fungal EMRE is necessary to activate animal-like MCU. However, the chimeric hsEMRE with the C-terminal of fungal EMRE (hsEMREct), is able to activate Hs-MCU, but is unable to reconstitute the mito-Ca2+ uptake when co-expressed with animal-like MCU. This appears to be contradicting the hypothesis.

Response: We do not find the results contradicting, instead they perfectly complement the hypothesis that the C-terminal domain of the fungal EMRE is despensable for the function of Hs-EMRE and the reconstitution of mt-Ca^{2+} uptake by Hs-MCU, whereas it is necessary for the function of the fungal EMRE and the activation of animal-like MCU. Firstly, we show that Hs-MCU is able to reconstitute mt-Ca^{2+} activity when expressed with either Hs-EMRE or fungal EMRE. This suggests that the molecular basis of Hs-MCU/Hs-EMRE functional interdependence lies within the functional domains that are conserved between fungal and human EMREs. It is therefore expected that the addition of the C-terminal domain of the fungal EMRE to Hs-EMRE does not affect the reconstitution of mt-Ca^{2+} uptake by Hs-MCU (thus it is not necessary). Instead, animal-like MCUs were unable to reconstitute mt-Ca^{2+} activity with either Hs-EMRE or Hs-EMREct, suggesting that the C-terminal domain is necessary but not sufficient for the activation of animal-like MCUs.

The authors should perform additional experiments to validate the involvement of the fungal C-terminal domain in MCU activation. There is no biochemical evidence presented for the animal-like MCU, fungal EMRE and fungal MICU1 interactions beyond the aequorin reporter.

Response: We agree that biochemical analysis of their physical interaction would be a nice addition to the measurement of Ca^{2+} kinetics in these strains. However, we believe that a reconstitution of Ca^{2+} uptake by animal-like MCUs and fungal EMREs provides the strongest evidence of a functional (and indirectly a physical) interaction. Indeed, a co-immunoprecipitation assay would simply show that animal-like MCUs and fungal EMREs interact but not that this interaction is functional. Instead, the observation of mt-Ca^{2+} uptake in yeast mitohcondria that only express those components is sufficient on its own to prove that the two proteins are functionally dependent.

5. In fungal EMRE, the MCU interacting domain GXXXA/S/G and the poly aspartate tail necessary for binding to MICU1 are fully conserved. Does hsEMREct still interact with fungal MCU and MICU1? These concerns can be easily addressed by co-immunoprecipitation experiments.

Response: The experiment suggested by the reviewer tries to address whether the C-terminal domain of the fungal EMRE is required for interacting with MCU or the known MCU interacting domain of Hs-MCU is sufficient for this. Of course it would be an interesting experiment, but not of substantial importance to support the overall claims of our study.

Minor:
1. Abbreviations should be mentioned in figure legends for all groups like “NI” and “pLKO” in Extended Data Fig. 7 is defined anywhere.

Response: Thank you for noting this. We now spell out all abbreviations used.

Response to reviewer #6:

General Comments: This is an important and novel study showing that previously identified animal and fungal MCUs, the targets of several structural and functional efforts, represent two distinct paralogous subfamilies originating from an ancestral duplication. The authors further show a complete “animal like” uniporter complex within chytrid fungi, including bona-fide orthologs of MCU, MICU1, and EMRE. This is a very important study for understanding the regulation of MCU. It is generally acknowledged that EMRE is only present in metazoans. Furthermore this study calls into question whether recent structural studies of fungal MCU (which this study suggested do not transport calcium) are an appropriate model to understand MCU mediated calcium transport in metazoans. This is a well done study and the authors have clearly addressed all the concerns raised in the previous review.

Response: We thank the reviewer for these nice comments. .

Specific Comments:
1. Fig 3b and 3c are very interesting. It might be nice to more explicitly in the text discuss that there are 2 EMRE genes in A. macrogynus (and indeed C. anguillulae in Fig 4b, though no data is shown for that species), and interestingly yeast reconstituted with the presence of MCU and each EMRE exhibit different degrees of calcium uptake (Fig. 3c).

Response: We now note and discuss this.

2. Fig 5 is great for clarifying the main conclusion; however, lines 103-108 make it sound as though the common ancestor of opisthokonts had both animal-like and fungal-specific MCUs and then Holozoa and fungi each lost one and kept the other. Fig. 5 makes it look like the animal-like MCU came first and then later the fungi had a duplication event. Can these be reconciled?

Response: We thank the reviewer for her/his observation. Our intention in the schematic representation was to present the taxonomic range that the various components are found across eukaryotes. However, we agree that the schema was potentially misleading. We have corrected Fig. 5 accordingly, and more extensively explained it in the legend.

Minor:
Based on how it's spelled everywhere else, "chytrid" is spelled wrong on line 116.

Response: Corrected.

Line 144 and 146 - it might be clearer to refer to "chytrid" EMRE instead of "fungal" EMRE just to be more specific since the story suggests that most fungi lost "animal-like" MCU and EMRE, but up to the authors

Response: We changed as suggested.

Line 159 - saying "the presence of the extra C-terminal domain in Hs-EMRE" is confusing, please clarify that the extra chytrid C-terminal domain was fused to Hs-EMRE

Response: We have now clarified this sentence.
